# Luminescent bis(benzo[*d*]thiazolyl)quinoxaline: facile synthesis, nucleic acid and protein BSA interaction, live-cell imaging, biopharmaceutical research and cancer theranostic application†

**DOI:** 10.1039/c9ra01498e

**Published:** 2019-03-18

**Authors:** Lavanya Thilak Babu, Gajanan Raosaheb Jadhav, Priyankar Paira

**Affiliations:** Department of Chemistry, School of Advanced Sciences, Vellore Institute of Technology Vellore-632014 Tamilnadu India priyankar.paira@vit.ac.in +91-416-2243092 +91-416-2243091; Drug Metabolism and Pharmacokinetics, Eurofins Advinus Ltd. 21 & 22, Phase II, Peenya Industrial Area Bangalore 560058 India

## Abstract

A series of quinoxaline-2-hydroxyphenylbenzothiazole scaffolds were synthesized and characterized using NMR, UV, fluorescence spectroscopy and LCMS. These newly synthesized compounds were found to be cytotoxic in human epithelioid cervix carcinoma (HeLa) and human colon cancer cell lines (Caco-2). Selectivity of the compounds 7e and 7g are more than 9 fold higher in Caco-2 cells with respect to the normal cell line HEK-293. The most fluorescent compound 7e has displayed high cytoselectivity, significant cellular uptake in HeLa cells and strong binding efficacy with DNA and BSA. The most potent compound 7g has primarily classified as BCS class 4 and BDDCS class 4.

## Abstract 2

A series of luminescent bis(benzo[*d*]thiazolyl)quinoxalines have been synthesized and their fluorescence properties, anticancer potency, DNA and BSA interactions, cellular uptake, and metabolic stabilities are investigated.

## Graphical abstract

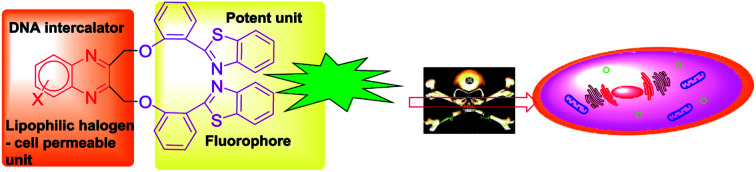

Cancer is a leading cause of morbidity and mortality worldwide. Since it is intricate in nature it requires a multi-step process for diagnosis and treatment.^1^ This impediment can be sorted out by theranostic drugs by which diagnosis and therapy are combined and implemented in a single agent. This facilitates the treatment as well as real-time monitoring of cells.^2,3^ Heterocyclic drugs have voluminous biomedical applications.^4^ The drugs that are available for cancer treatment consist of one or more heterocyclic rings. Quinoxalines are a significant class among the heterocyclic drugs. They have attracted attention in pharmacology due to their wide scope in biological applications including as a well-known DNA intercalator.^5^ Quinoxaline is the key chemical component of many antibiotics such as echinomycin, bleomycin, actinomycin and clofazimine which are known to inhibit several Gram-positive bacteria and are also active towards various tumours.^6–8^ Various anticancer drugs that incorporate quinoxaline as the core moiety had exhibited activity against solid tumours and are applicable in clinical trials.^9,10^

Similarly, benzothiazole possesses anticancer, antimicrobial, antidiabetic, anticonvulsant, anti-inflammatory, antiviral and anti-tubercular activities.^11^ Benzothiazole conjugated compounds exhibited high potency in various cancer cell lines.^12^ These compounds exhibited as a chelating metal ion. It chelates metal ion that is present in amyloids and it also arrests the accumulation of metals in amyloid fibrils.^13,14^ Extended derivations of benzothiazoles are viable and it is applicable for cancer theranostics. For example, [Gd(DO3A-BTA)(H_2_O)] a theranostic agent is employed in tumour specificity, confirmed by tracking MR images in cytosol and nuclei of MDA-MB-231, MCF-7, and SK-HEP-1 cancer cell lines.^15^ Recently our group has reported novel benzothiazole–quinoline conjugates as cancer theranostics.^16^ Furthermore, this type of conjugates is also developed as a novel human A_3_ receptor antagonist.^17^ Moreover, benzothiazole conjugation with quinolones was applicable in sensing of Hg^2+^ in HeLa cells by fluorescence turnover.^18^ Novel fluorescent conjugates of 2-(benzothiazole-2-yl)-phenol (BTP) were also used in the selective detection of superoxide anions by ESIPT.^19^ Several benzothiazole conjugates were also reported in H_2_O_2_-responsive detection *via* theranostic probe for detection of endothelial injury.^20^ Nowadays, scientists are more interested to design organic molecules which specifically target the proteins or nucleic acids like DNA to understand the drug metabolism, absorption, excretion and distribution and for getting a better perception of DNA–protein interactions.^21–24^ In view of the significance of quinoxaline and benzothiazole in drug discovery, we have intrigued our attention on developing a novel pharmacophore in a single domain for cancer theranostic application (Fig. 1). Herein, we have developed a series of substituted quinoxaline in conjugation with well-known fluorophore 2-hydroxyphenylbenzothiazole by adopting an efficient methodology and their *in vitro* studies like DNA, BSA binding, cytotoxicity study, permeability, stability, and solubility study were done in detail.

**Fig. 1:**
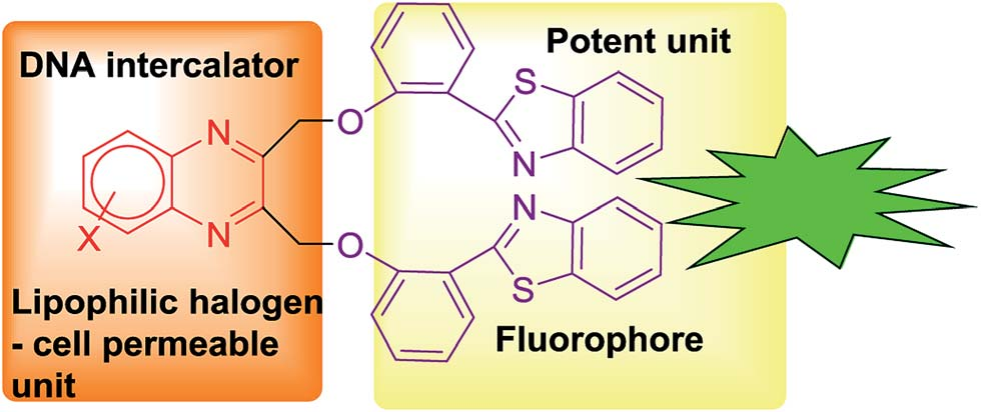
Design of bis(benzo[*d*]thiazolyl)quinoxaline scaffold.

Initially, an equal mole of 2-aminothiophenol (1) and 2-hydroxybenzaldehyde (2) were dissolved in ethanol and an adequate amount of silica gel was added to the mixture to prepare the slurry. The slurry was then air dried and subjected to a microwave oven at 490 watt (120 °C) for 15 min. The reaction was monitored by TLC using hexane/ethyl acetate (3 : 1) solvent system. After completion of the reaction, ethyl acetate was added to the solid support and the product was recovered by filtering the solution from the resin by Whatman filter paper. The solution is then transferred to the beaker and air dried. Subsequently, the solvent was reduced gradually and white needle-like crystals of benzothiazolylphenol (BTP) (3) was obtained with high yield (Scheme 1). Compound 3 was fully characterized by ^1^H-NMR and ^13^C-NMR spectroscopy. The characteristic singlet OH peak at downfield around *δ* 12.56 ppm and aryl CH protons at *δ* 6.9–8.0 ppm was observed in ^1^H-NMR.^16^ Similarly, another precursor quinoxaline dibromides (6a–g) were synthesized by dissolving the equivalent amount of 1,4-dibromobutane-2,3-dione (5) and phenylene-1,2-diamines (4a–g) in DCM stirring for 2 h at ambient temperature. The formation of the products with high yield was confirmed by TLC using hexane/ethyl acetate (3 : 1) solvent system. The solvent was evaporated and square shaped crystals of quinoxaline dibromide were obtained with high yield. The structures were confirmed by ^1^H-NMR spectroscopy. In ^1^H NMR the characteristic singlet methylene protons of compounds (6a–g) were observed in the upfield region at *δ* 4.9 ppm. The aromatic peaks of the quinoxaline ring were obtained at *δ* 7.7–8.0 ppm range. When electronegative halogen groups are substituted in quinoxaline ring at R_1_ and R_2_ position those peaks are shifted towards the more downfield region. To synthesize the quinoxaline–BTP conjugates (7a–g) quinoxaline derivatives and BTP (1 : 2 ratio) were dissolved in acetone followed by the slurry preparation using basic alumina. It is then air dried and subjected to microwave at 490 watts (120 °C) for 30 min. The progress of the reaction was monitored eventually by TLC using hexane/ethyl acetate (3 : 1) solvent system. After completion of the reaction, the compound was recovered by adding ethyl acetate to it and the alumina is filtered off using Whatman filter paper. The fine needle like crystals of compound 7a–7g was obtained by slow evaporation of ethylacetate (Scheme 1).

**Scheme 1:**
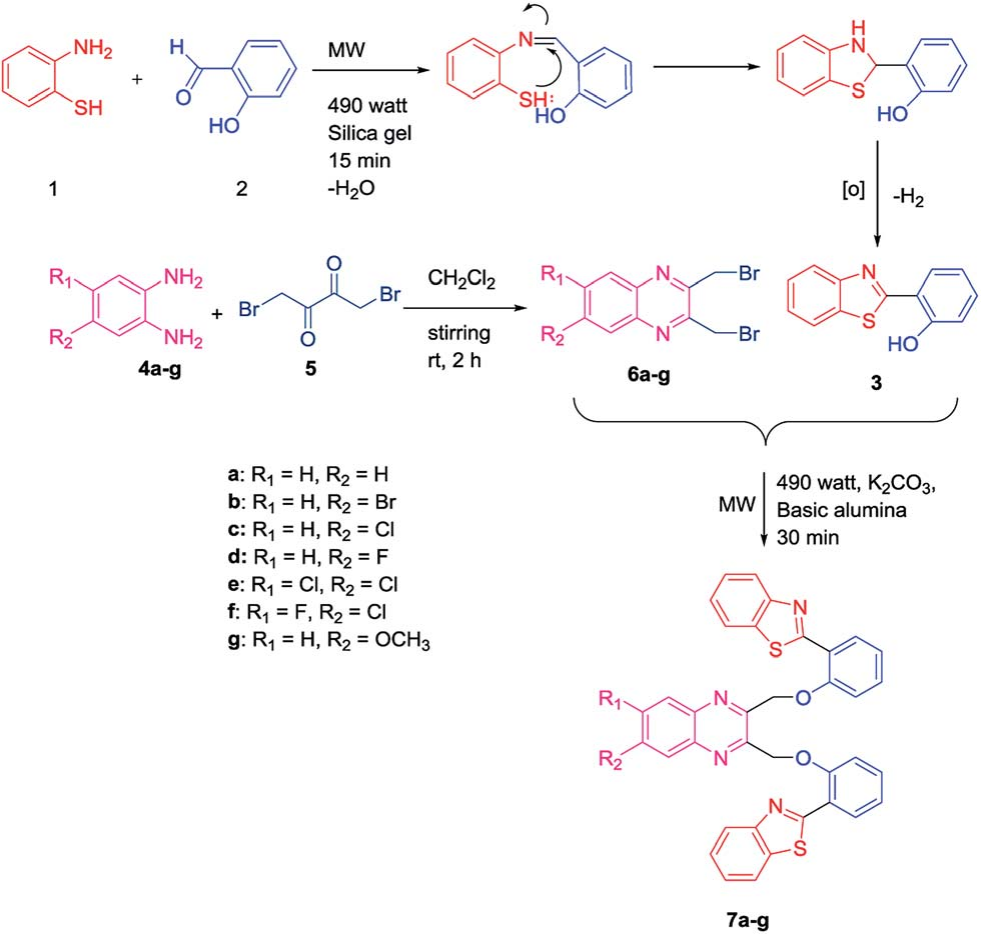
Synthetic scheme for bis(benzo[*d*]thiazolyl)quinoxaline analogues.

The formation of these products was further confirmed by ^1^H NMR, ^13^C-NMR and mass spectra. The characteristic singlet peak of aliphatic CH_2_ protons was observed in the range of *δ* 5–6 ppm. Undoubtedly, the singlet peak of –OH was not found in the ^1^H NMR. In ^13^C NMR the characteristic CH_2_ peak is observed at *δ* 71 ppm. In the mass spectra, the characteristic (M + H)^+^ peaks of the compounds (7a–g) were observed which corresponded their molecular mass. The halogen groups containing compounds (7b, 7c, 7e, 7f) exhibited characteristic isotopic patterns in their mass spectra.

To know the absorption and emission behaviour of these synthesized compounds (7a–g), a standard concentration (3 × 10^−6^ mol l^−1^) in water has been used. In UV-vis spectra, *λ*_max_ of all these compounds are observed at 320–330 nm due to π–π* transition (Fig. S1a†). The corresponding emission peaks are observed at 400–500 nm (Fig. S1b†). The emission of quantum yield (*Φ*) was calculated for all of the compounds (7a–g) using eqn (1) (Table S1†). We found that compound 7d and 7e exhibited the highest quantum yields (0.04) among all the other scaffolds.

The stability of compounds in water, GSH, and MTT condition (10% DMSO in PBS buffer) was studied. Water is a key factor because it widens the application in the biological field. Mostly in cancer cells, GSH level are found higher and hence there are chances for cleavage of the compounds. The stability of complex 7e in water (pH 7.2) and GSH were investigated by UV-vis spectroscopy over a period of 20 h of time (Fig. S2a and b†). There were no changes in absorbance and *λ*_max_ was observed with time which designated the stability of compound 7e in water and GSH. Likewise, in MTT condition, an insignificant change of absorbance and *λ*_max_ correspond to the stability of this compound even after 18 h (Fig. S2c†).

DNA is one of the key target for cancer therapy. The drug molecules bind with the DNA by three major modes (i) electrostatic interactions with the DNA double helix (ii) binding to grooves of DNA (iii) intercalation between the base pairs of DNA.^25^ The titration in UV was performed by increasing concentration of DNA from 10–60 μM into a fixed drug concentration (20 μM). The intra-ligand absorption bands around 290–350 nm (π–π* transition) were used in observation of the interaction of DNA and compound 7e (Fig. S3a†). There was a hypochromic shift with a slight blue shift in the wavelength of compound 7e was observed which concluded that complex 7e bind to DNA through intercalative binding mode.^26^ Intrinsic binding constant *K*_b_ was calculated to be 6.9 × 10^3^ M^−1^ using the eqn (2) and Fig. S3b.† Ethidium bromide (EtBr) displacement assay is commonly used as a diagnostic technique to identify the intercalation ability of small molecules with DNA. Ethidium bromide, a nonspecific intercalator, gives a strong fluorescence while intercalating DNA base pairs. This enhanced fluorescence can be quenched by another chemical moiety which can competitively replace EtBr and binds to the same site.^27^ A solution containing DNA bound ethidium bromide displayed intense fluorescence at *λ* 608 nm when excited at 485 nm. The original fluorescence intensity of EtBr–DNA complex is rapidly quenched by increasing the concentration of compound 7e (Fig. S3c†). Stern–Volmer quenching constant (*K*_sv_), apparent binding constant (*K*_app_) and high intrinsic binding constant (*K*_b_) for compound 7e were tabulated (Table 1, eqn (3) and (4)†) which indicate the intercalative binding manner of this compound with DNA.

**Table 1:** DNA-binding parameter for compound 7e with CT-DNA

Compound	*K*_b_ a (M^−1^)	% Hypochromism^b^	*K*_sv_ c (M^−1^)	*K*_app_ d (M^−1^)
7e	6.9 × 10^3^	50	5.5 × 10^3^	5.7 × 10^5^

a *K*_b_, intrinsic DNA binding constant from UV-visible absorption titration.

b *K*_sv_, Stern–Volmer quenching constant.

c *K*_app_, apparent DNA binding constant from competitive displacement from fluorescence spectroscopy.

d Apparent binding constant.

Serum albumin has the capacity to bind with the ligands and helps in the uptake of the drug inside the cells. BSA is commonly used in this study because of its similar structural homology to HSA. Higher the binding strength of the molecules with BSA increases their half-life and hence the renal clearance also decreases. BSA molecule shows intrinsic fluorescence due to the presence of aromatic amino acids like tyrosine, phenylalanine, mainly tryptophan in its quaternary structure. A significant quenching of BSA fluorescence can be observed upon interaction with drug molecule.^27^ The BSA solution of 3 μM concentration was titrated against 10–100 μM of the drug 7e upon excitation of 280 nm. Fig. S4a† clearly indicates that a considerable quenching (more than 50%) of BSA occurs with an increase in compound 7e concentration. Interestingly, a distinct emission peak at 410 nm with a sharp isosbestic point at 390 nm arises in a high concentration of the drug, indicates a strong interaction of compound 7e with BSA. The Stern–Volmer constant (*K*_BSA_), quenching constant (*K*_q_), binding constant (*K*), a number of binding sites (*n*) were calculated from the eqn (5) and (6) (Fig. S4b and c†). The *K*_q_ values of the newly synthesized compounds were in the order of 10^12^ M^−1^ s^−1^ which were higher enough than maximum scatter collision quenching constant (2.0 × 10^10^ M^−1^ s^−1^) of BSA quenchers (Table 2).^28^ High potency of these complexes in cancer cells may be attributed by the strong binding with serum albumin which overcomes the drug resistance by GSH.

**Table 2:** BSA binding data for compound 7e

Compound	*K*_BSA_ a (M^−1^)	*K*_q_ b (M^−1^ s^−1^)	*K* c (M^−1^)	*n* d
7e	1.02 × 10^4^	1.02 × 10^12^	2.62 × 10^2^	1.87

a *K*_BSA_, Stern–Volmer quenching constant.

b *K*_q_, quenching rate constant.

c *K*, binding constant with BSA.

d *n*, the number of binding sites.

Cytotoxicity studies were performed for all the synthesized quinoxaline compounds (7a–7g) using standard 3-(4,5-dimethylthiazol-2-yl)-2,5-diphenyltetrazolium bromide (MTT) assay beside a panel of cancer cell lines such as human epithelioid cervix carcinoma (HeLa), human colorectal adenocarcinoma cell line (Caco-2) and one normal human embryonic kidney cells (HEK-293) in triplicates (Table 3). While the synthesized compounds exhibited moderate activity, but these showed significant selectivity in both the cancer cells with respect to normal kidney cell. It is noteworthy to mention that Compound 7a and 7g presented best cytoselectivity in HeLa and Caco-2 cell lines respectively.

**Table 3:** Preliminary MTT cytotoxicity screening of bis(benzo[*d*]thiazolyl)quinoxaline scaffolds (7a–g) at 24–72 h of drug exposure

Compound	Cell lines				
	IC_50_ (μM)^a^			SF^b^	
	Caco-2^c^	HeLa^d^	HEK 293^d^	Caco-2	HeLa
7a	69.8 ± 0.8	73.1 ± 1.7	120 ± 0.9	1.71	1.6
7b	109 ± 0.8	>100	140 ± 1.4	1.28	1.4
7c	75 ± 3.5	68.61 ± 0.2	160 ± 2.1	2.13	2.3
7d	92 ± 4.9	117 ± 5.4	>200	>2.17	>1.7
7e	20.9 ± 1.6	38.9 ± 0.1	>200	>9.56	>5.1
7f	>100	44.3 ± 0.3	>200	>2	>4.5
7g	20.8 ± 1.6	36.8 ± 0.2	>200	>9.6	>5.4
Cisplatin	21.2 ± 1.6	14.5 ± 0.8	50 ± 1.2	2.4	3.4

a IC_50_ is the concentration of the synthesized quinoxaline compounds and cisplatin at which 50% of cells undergo cytotoxic cell death under treatment.

b SF (selectivity factor) = ratio of IC_50_ for HEK-293 to IC_50_ for all the cancer cell lines.

c 72 h incubation.

d 24 h incubation.

Live cell imaging was performed in HeLa cell line. Compound 7e in 50 μM concentration in the buffer, were incubated with HeLa cells for 4 h. Subsequently, the treated HeLa cells were excited under green filters in the fluorescence microscopy and we observed a significant red fluorescence from the drug-treated cells (Fig. 2). Significant potency, selectivity and cellular imaging property of compound 7e, mark it as a theranostic agent.

**Fig. 2:**
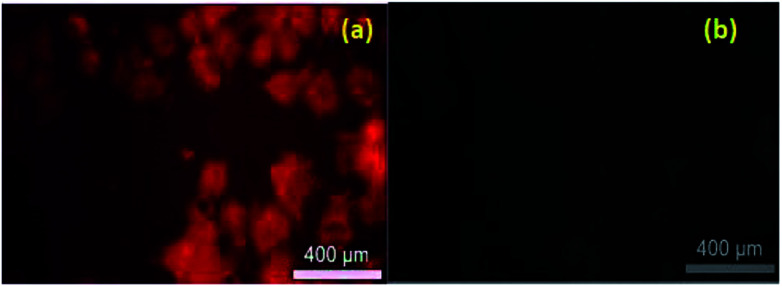
Fluorescence and bright-field images of live cells: (a) fluorescent image of HeLa cell with compound 7e (50 μM in PBS buffer); incubation time 2 hours (b) bright-field image of HeLa cell with compound 7e (50 μM in PBS buffer); incubation time 2 hours. Scale bar 100 μm.

LC-MS/MS methods were developed for compound 7g to quantify the drug concentration and *in vitro* study (*i.e.* buffer solubility, buffer stability, metabolic stability, and permeability) in a systemic manner (Table 4). Chromatographic elution conditions were set with the use of 5 mM ammonium formate containing 0.1% formic acid and acetonitrile containing 0.1% formic acid with C8, Kromasil, and 4.6 × 50 mM, 5 μM as a stationary phase (Fig. S5a and b†). Compound 7g was subjected to solubility assessment, through incubation with buffer for 2 h. For the data analysis, % accuracy of compound 7g was calculated and it was soluble up to 25 μM (Fig. 3, eqn (7)†).

**Table 4:** Stability study of compound 7g in different concentration

Stability of Compound 7g	
Concentration (μM)	% Remaining in 120 min
3.13	59
6.25	68
12.50	81
25.00	110
50.00	110
100.00	71

**Fig. 3:**
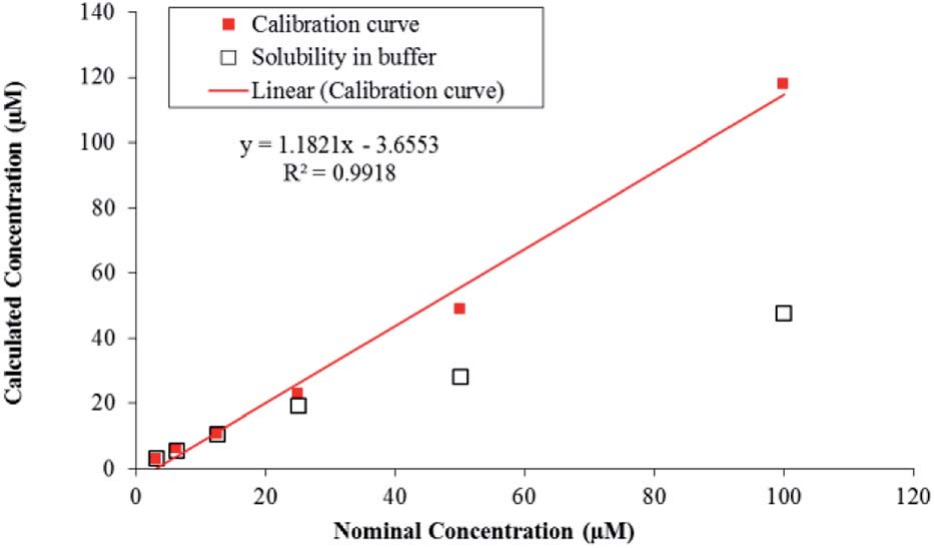
Solubility of compound 7g in pH 7.4 buffer.

The *in vitro* solubility and stability of 7g were evaluated in pH 7.4 buffer. The buffer solubility and stability studies were conducted at six different concentrations ranging from 3.13 to 100 μM. For the data analysis, % stability of compound 7g at 120 min was calculated with respect to 0 min time point samples and it was stable up to 2 hours in pH 7.4 buffers. Data analysis was performed through the eqn (8)† and was tabulated (Table 4). Compound 7g found to be stable up to 2 hours with % remaining of 71% in pH 7.4.

Compound 7g permeation ability (*i.e.* permeability) was assessed through Caco-2 cell monolayer. Permeability study was planned in both directions from apical to basolateral (A to B) and basolateral to apical (B to A) side. A to B is for checking drug permeation characteristics from the intestine (site of absorption) to systemic circulation and B to A is for checking drug efflux back to the intestine. Compound 7g has shown very low concentration (*i.e.* below the lower limit of quantitation) in samples because of low permeability across Caco-2 cell monolayer in the tested conditions (Table 5).

**Table 5:** Compound 7g Caco-2 permeability study

Compound 7g Caco-2 permeability results summary		
Compound	Compound 7g	
Concentration (μM)	10	
Transport	A to B	B to A
*P*_app_ (nm s^−1^)	—	—
Efflux ratio	—	
% Recovery	101	96
Replicate	*N* = 3	*N* = 3

The *in vitro* metabolic rate of 7g was determined in liver microsomes and the intrinsic clearance (CLintr) was estimated. The study was conducted at 0.5 μM substrate concentration and microsomal protein concentration of 0.5 mg ml^−1^. Metabolic stability study in the presence of microsomes and cofactor NADPH was performed at 37 °C, 60 rpm for 30 min time duration. NADPH free control incubation was performed simultaneously for 30 min to understand if there are any non-enzymatic metabolism related issues. Compound 7g was observed to be more stable in rat and dog liver microsomes than the other two species in the tested conditions (Fig. S6,†Table 6). Intrinsic clearance as given in Table 6, was calculated using eqn (8).†

**Table 6:** Metabolic stability of compound 7g

Compound	Matrix	% Metabolism in 30 min	Half life (min)	CL_int_ (μl per min per mg protein)
Compound 7g	MLM set 1	12	109.80	13
	MLM set 2	37	49.10	28
	RLM set 1	12	351.00	4
	RLM set 2	19	101.20	14
	DLM set 1	21	77.05	18
	DLM set 2	14	114.30	12
	HLM set 1	35	49.26	28
	HLM set 2	38	55.90	25

## Conclusions

In summary, we have developed a convenient protocol for the synthesis of quinoxaline-2-hydroxylphenylbenzothiazole complexes. Most of the compounds reported here displayed moderate potency and selectivity in Caco-2 and Hela cell lines. Compound 7e and 7g exhibited best cytoselective profiles in all the cancer cells with respect to the normal cell. These compounds also showed better efficacy than cisplatin in the Caco-2 cell line. Compound 7g exhibited good buffer stability and metabolic stability in microsomes. Significant cellular uptake of compound 7e in HeLa cells was also observed in the fluorescence microscope. Eventually, compound 7e was recognized as the most potent fluorescent organic molecule for cancer theranostic application.

## Conflicts of interest

There are no conflicts to declare.

## Supplementary Material

RA-009-C9RA01498E-s001
