# Supplementary material for: Luminescent bis(benzo[d]thiazolyl)quinoxaline: facile synthesis, nucleic acid and protein BSA interaction, live-cell imaging, biopharmaceutical research and cancer theranostic application
Source: RSC Adv. 2019 Mar 18;9(16):8748–52. doi: 10.1039/c9ra01498e (PMC9061853; doi:10.1039/c9ra01498e)
Supplement: RA-009-C9RA01498E-s001 [file RA-009-C9RA01498E-s001.pdf]

Electronic Supplementary Material (ESI) for RSC Advances.
This journal is © The Royal Society of Chemistry 2019

Supporting information

Luminescent bis(benzo[d]thiazolyl)quinoxaline: Facile synthesis, Nucleic Acid and protein BSA interaction, Live-cell imaging and Cancer theranostic application

Lavanya Thilak Babu†, Gajanan Raosaheb Jadhav† and Priyankar Paira

Contents:

Experimental section 2-10

Figure of NMR, LCMS, and IR spectra of synthesized compounds 11-34

UV and Fluorescence study 35

Stability study 36

DNA binding study 37

BSA binding study 38

*Chromatogram for compound **7e*** 38-39

Metabolic stability study 40-41

Experimental section:

2.1. Materials and methods

The chemicals 2 amino benzothiazol, 2 hydroxy benzaldehyde, Benzene 1,2 diamine, 4,5 dichloro-o-phenylene diamine, 4-bromo-o-phenylene diamine, 4-fluro-o-phenylene diamine,4-Methoxy-o-phenylene diamine, 4-chloro-o-phenylene diamine, 4-chloro-5-Fluro-o-phenylene diamine, 1,4 dibromo 2,3 butane dione were purchased from Avra chemical limited. CT-DNA, ethidium bromide, bovine serum albumin (BSA) were purchased from Himedia Chemical Limited. All the organic solvents used for chemical synthesis were procured from E. Merck (India) of analytical grade. HeLa, Caco-2 and MRC-5 cell lines were purchased from NCCS, Pune. ^{1}HNMR and ^{13}CNMR spectra were analysed using a Bruker DPX spectrometer at 400 MHz and 100 MHz respectively using tetramethylsilane as internal standard and the chemical shifts are reported in ppm units. Abbreviations are as follows: s, singlet; d, doublet; dd, double doublet; t, triplet; m, multiplet. CATA-R Microwave set up has been used for the reaction. The melting points of PBT-quinoxaline series were measured by Elchem Microprocessor based DT apparatus using open capillary tubes and are uncorrected. The synthesized compounds were also characterized by Schimadzu LCMS-4000 LC-MS instrument, having 4000 triple quadrapole MS, using Methanol as the solvent. Thin layer chromatography was performed on pre-coated silica gel 60 F_{254} aluminum sheets (E. Merck, Germany) using the solvent system in hexane/Ethyl Acetate (3:1) mixture and the spots were identified when exposed to UV light. The UV-Visible spectrum was recorded by UV-2550 instrument, Shimadzu Corporation, and Kyoto, Japan. The fluorescence spectra were analysed on Hitachi F-7000 FL spectrophotometer. Elisa reader and 96-well plate has been used for MTT assay. Fluorescence imaging study was performed using Olympus model CKX41 microscope. The melting points were determined using capillary melting point apparatus.

*2.2. General procedure for the synthesis of 2,3-bis (bromomethyl quinioxaline)series (**6a-6g**)*

Benzene 1,2 diamine (**4a-g**) (100 mg) and 1,4-dibromobutane-2,3-dione (**5**) (108.14mg) were taken in 50 ml round bottom flask and dissolved completely in dichloromethane (DCM) followed by stirring for 2 h at ambient temperature. The progress of reaction was monitored by TLC using hexane/ethyl acetate (3:1) solvent system. After completion of reaction the solvent DCM was evaporated and the white needle like crystals of 2,3-bis bromomethyl quinioxalines (**6a-g**) were formed with 90-95% yield.

*2.3. Characterization of 2,3-bis (bromomethyl quinioxaline)series (**6a-g**)*

2,3-bis(bromomethyl)quinoxaline (**6a**): ^{1}H NMR (400 MHz, $CDCl_3$): σ 8.08-8.05 (m, 2H), 7.80-7.77 (m, 2H), 4.93 (s, 4H).

6-bromo-2,3-bis(bromomethyl)quinoxaline (**6b**): ^{1}H NMR (400, MHz $CDCl_3$): σ 8.25 (s, 1H), 7.92 (d, *J* = 8.8 Hz, 1H), 7.85 (d, *J* = 8.8 Hz, 1H), 4.89 (s, 4H).

2,3-bis(bromomethyl)-6-chloroquinoxaline (**6c**): ^{1}H NMR (400, MHz $CDCl_3$): σ 8.06 (S, 1H), 8.00 (d, *J* = 9.2 Hz, 1H), 7.73 (d, *J* = 9.2 Hz, 1H), 4.89 (s, 4H).

2,3-bis(bromomethyl)-6-fluoroquinoxaline (**6d**): ^{1}H NMR (400, MHz, $CDCl_3$): σ 8.10-8.06 (m, 1H), 7.71-7.68 (m, 1H), 7.60-7.55 (m, 1H), 4.92 (s, 2H), 4.90 (s, 2H).

2,3-bis(bromomethyl)-6,7-dichloroquinoxaline (**6e**): ^{1}H NMR (400 MHz, $CDCl_3$): σ 8.19 (s, 2H) ,4.88 (s, 4H).

2,3-bis(bromomethyl)-6-chloro-7-fluoroquinoxaline (**6f**): ^{1}H NMR (400 MHz, $CDCl_3$): σ 8.16 (d, *J* = 7.52 Hz, 1H), 7.79 (d, *J* = 9Hz, 1H), 4.88 (s, 4H).

2,3-bis(bromomethyl)-6-methoxyquinoxaline (**6g**): ^{1}H NMR (400 MHz, $CDCl_3$): σ 7.94 (d, *J* = 9.2 Hz, 1H), 7.42-7.45 (m, 1H), 7.35 (s, 1H), 4.90 (d, 2H), 4.89 (d, 2H), 3.98 (s, 3H).

*2.4. Synthesis and characterization of 2-benzothiazolyl phenol (BTP) (**3**)*

The equal molar of 2-aminothiophenol (**1**) and 2-hydroxybenzaldehyde (**2**) was dissolved in ethanol and an adequate amount of silica gel was added to make the slurry. The prepared slurry was further air dried in vacuum followed by microwave irradiation at 490 watt for 15 min. The progress of reaction was monitored by TLC using hexane/ethyl acetate (3:1) solvent system. After completion of the reaction, ethyl acetate was added to the solid support and the product was recovered by filtering the solution from the resin by whattman filter paper. The solution is then transferred to the beaker and air dried. Subsequently, the solvent was reduced gradually and white needle-like crystals of benzothiazolylphenol (BTP) (**3**) was obtained with high yield.

2-(benzo[d]thiazol-2-yl)phenol (3)[1]: ^{1}H NMR (400 MHz, $CDCl_3$) σ 7.99 (d, *J* = 8 Hz, 1H), 7.90 (d, *J* = 8.0 Hz, 1H), 7.70 (d, J = 8.0 Hz, 1H), 7.51 (t, *J* = 8.0 Hz, 1H), 7.43-7.37 (m, 2H), 7.11 (d, *J* = 8.0 Hz, 1H), 6.96 (t, *J* = 7.6 Hz, 2H); ^{13}C NMR ($CDCl_3$, 100 MHz); 116.8, 117.8, 119.5, 121.5, 122.2, 125.5, 126.7, 128.4, 132.6, 132.7, 151.8, 157.9, 169.32.

*2.5. General procedure for the synthesis of BTP-Quinoxaline series (7**a**-7**g**)*

2,3-bis (bromomethyl quinioxaline) series (**6a-6g**) and BTP (1:2 ratio) were dissolved in acetone followed by the slurry preparation using adequate amount basic alumina. It is noteworthy to mention that 1.1 equivalents of K_2CO_3 were added to the reaction mixture for speed up the reaction. It is then air dried and subjected to microwave at 490 watts (120° C) for 30 min. The progress of the reaction was monitored eventually by TLC using hexane/ethyl acetate (3:1) solvent system. After completion of the reaction, the compound was recovered by adding ethyl acetate to it and the alumina is filtered off using whattman filter paper. The fine needle like crystals of compound **7a-7g** was obtained by slow evaporation of ethylacetate with high yield.

*2.6. Characterization of BTP-Quinoxaline series (7**a**-**g**)*

*2,3-bis((2-(benzo[d]thiazol-2-yl)phenoxy)methyl)quinoxaline (7**a**)* : mp: 130-135 °C; R_f: 0.78; yield: 82 %; ^{1}H NMR (400 MHz, $CDCl_3$): σ 8.49 (dd, J_1 = 7.6 Hz, J_2 = 1.6 Hz, 2H), 8.22 (dd, J_1 = 6.4 Hz, J_2 = 3.6 Hz, 2H), 8.06 (d, J = 8.0 Hz, 2H), 7.89 (dd, J_1 = 6.4 Hz, J_2 = 3.6 Hz, 2H), 7.68 (d, J = 8 hz, 2H), 7.46 (t, J = 8.0 Hz, 2H), 7.33-7.29 (m, 4H), 7.17 (d, J = 8.0 Hz, 2H), 7.09 (t, J =7.6 Hz, 2H), 5.88 (s, 4H, CH_2); ^{13}C NMR ($CDCl_3$, 100 MHz): σ 71.2 (CH_2), 113.0, 121.3, 121.8, 122.4, 122.8, 124.6, 126.0, 129.3, 129.8, 130.9, 131.8, 135.7, 141.5, 150.5, 152.1, 156.0, 162.6; IR (KBr, cm^{-1}): 2978.1, 1597.1, 1450.5, 1114.9, 748.4; LC-MS (MeOH) for $C_{36}H_{24}N_4O_2S_2$ $[M+H]^+$: 609.14, found: 609.0.

2,2'-((((6-bromoquinoxaline 2,3diyl)bis(methylene))bis(oxy))bis(2,1phenylene))bis(benzo[d]thiazole) (7**b**) : mp: 145°C; R_f: 0.75; HPLC purity (84.0%, eluent: 70% MeOH/ACN, Rt: 0.33 min); ^{1}H NMR (400 MHz, $CDCl_3$): σ 8.49-8.47 (m, 2H), 8.40 (s , 1H), 8.07 (d, J = 8.8, 2H), 7.96 (d, J = 2 Hz, 1H), 7.69 (d, J = 7.6 Hz , 2H) 7.49-7.45 (m, 2H), 7.35-7.29 (m, 4H), 7.16-7.13 (m, 2H), 7.11 (d, J = 7.6 Hz, 2H), 5.86 (d, J =8 Hz, 4H); ^{13}C NMR ($CDCl_3$, 100 MHz): σ 71.0 (CH_2), 113.0, 121.3, 121.8, 122.4, 122.8, 124.6, 126.0, 129.3, 129.8, 130.9, 131.8, 135.7, 141.5, 150.5, 152.1, 156.0, 162.6; IR (KBr, cm^{-1}): 1597.1, 1429.2, 1290.4, 1116.8, 750.3, 428.2; LC-MS (MeOH) for $C_{36}H_{24}BrN_4O_2S_2$ $[M+H]^+$

Calcd: 687.05, found: 687, $[M+H]^+$, 689 $[M+2+H]^+$.

*2,2'-((((6-chloroquinoxaline-2,3-diyl)bis(methylene))bis(oxy))bis(2,1-phenylene))bis(benzo[d]thiazole) (7**c**)* : mp: 150°C; R_f: 0.74; HPLC purity (96.3%, eluent: 70% MeOH/ACN, Rt: 0.34 min); ^{1}H NMR (400 MHz, $CDCl_3$): σ 7.82 (d, *J* = 7.6, 2H), 7.54 (s, 1H), 7.48 (d, *J* = 8.8 Hz , 1H), 7.40 (d, *J* = 8.4 Hz, 2H), 6.81 (t, *J* = 7.6 Hz, 2H), 6.68-6.59 (m, 4H), 6.49-6.46 (m, 4H) 5.19 (d, *J* = 5.2 Hz, 4H); ^{13}C NMR ($CDCl_3$, 100 MHz): σ 71.0 (CH_2), 112.9, 117.8, 119.5, 121.3, 121.9, 122.2, 122.5, 122.9, 124.7,125.5, 126.0, 126.7, 128.2, 128.4, 129.9, 130.5, 131.8, 131.9, 132.6, 132.7, 135.7, 136.8, 139.9, 141.7, 150.6, 151.5, 152.1, 155.9, 162.6; IR (KBr, cm^{-1}): 1579.7, 1498.7, 1290.4, 964.4, 750.3; LC-MS (MeOH) for $C_{36}H_{24}ClN_4O_2S_2$ $[M+H]^+$ Calcd: 643.10, found: 643.0 $[M+H]^+$

*2,2'-((((6-fluoroquinoxaline-2,3-diyl)bis(methylene))bis(oxy))bis(2,1-phenylene))bis(benzo[d]thiazole) (7**d**)*: mp: 135-139°C; R_f: 0.5; HPLC purity (93.0%, eluent: 70% MeOH/ACN, Rt: 0.35 min); ^{1}H NMR (400 MHz, $CDCl_3$): σ 8.48 (d, *J* = 8 Hz, 2H), 8.22- 8.19 (m, 1H), 8.15 (d, *J* = 8.4 Hz, 2H), 7.83 (dd, J_1 = 9.2 Hz, J_2 = 2.8 Hz, 1H), 7.67 (d, *J* = 8 Hz, 3H), 7.50-7.47 (t, *J* = 7.2 Hz, 2H), 7.06 (t, *J* = 7.6 Hz, 2 H), 5.89 (d, *J* = 6 Hz, 4H); ^{13}C NMR ($CDCl_3$, 100 MHz): σ 71.0 (CH_2), 112.8, 113.0, 121.3, 121.9, 122.6, 125.0, 126.3, 130.0, 131.4, 131.5, 132.2, 151.3, 156.0; IR (KBr, cm^{-1}): 1597.1, 1487.1, 1220.9, 754.2; LC-MS (MeOH) for $C_{36}H_{24}FN_4O_2S_2$ $[M+H]^+$ Calcd: 627.1, found: 627.0 $[M+H]^+$.

*2,2'-((((6,7-dichloroquinoxaline-2,3-diyl)bis(methylene))bis(oxy))bis(2,1-phenylene))bis(benzo[d]thiazole) (7**e**)*: mp: 145-150°C; R_f: 0.78; HPLC purity (88.0%, eluent: 70% MeOH/ACN, Rt: 0.91 min); ^{1}H NMR (400 MHz, $CDCl_3$): σ 8.47 (d, *J* = 7.2 Hz, 2H), 8.32 (s, 1H), 8.06 (d, *J* = 8 Hz, 2H), 7.69 (d, *J* = 8 Hz, 2 H), 7.47 (t, *J* = 7.6 Hz, 3H), 7.35-7.26 (m, 4H), 7.10 (t, *J* = 8.4 Hz, 4H), 5.84 (s, 4H); ^{13}C NMR ($CDCl_3$, 100 MHz): σ 70.8 (CH_2), 112.9, 121.3, 122.0, 122.5, 122.9, 124.8, 126.1, 129.9, 130.0, 131.8, 135.6, 135.7, 140.1, 151.6, 152.1, 155.8, 162.5; IR (KBr, cm^{-1}): 1585.5, 1450.5, 1213.2, 1111.0, 748.4, 426.27; LC-MS (MeOH) for $C_{36}H_{23}Cl_2N_4O_2S_2$ $[M+H]^+$ Calcd: 677.06, found: 677.0.

*2,2'-((((6-chloro-7-fluoroquinoxaline-2,3-diyl)bis(methylene))bis(oxy))bis(2,1-phenylene))bis(benzo[d]thiazole) (7**f**)*: mp: 155-160°C; R_f: 0.78; HPLC purity (91.0%, eluent: 70% MeOH/ACN, Rt: 0.33 min); ^{1}H NMR (400 MHz, $CDCl_3$): σ 8.47 (d, *J* = 7.56 Hz, 2H), 8.29 (d, *J* = 7.2 Hz, 1H), 8.06 (d, *J* = 8.0 Hz, 2H), 7.91 (d, *J* = 8.8 Hz, 1H), 7.69 (d, *J* = 8.0 Hz, 2H), 7.47 (m, 3H), 7.33 (m, 3H), 7.12 (m, 4H), 5.85 (s, 4H); ^{13}C NMR ($CDCl_3$, 100 MHz): σ 70.8 (CH_2), 113.0 (CH), 113.9 (CH), 121.2 (CH), 122.0 (CH), 122.5 (C), 122.9

(CH), 124.7 (CH), 126.0 (CH), 130.0 (CH), 130.6 (CH), 131.7 (CH), 135.7 (C), 151.6 (C), 152.2 (C), 155.8 (C), 162.5 (C); IR (KBr, cm^{-1}): 1585.5, 1467.8, 1201.6, 752.2; LC-MS (MeOH) for $C_{36}H_{23}ClFN_4O_2S_2$ $[M+H]^+$ Calcd: = 661.09, found: 660.8

*2,2'-((((6-methoxyquinoxaline-2,3-diyl)bis(methylene))bis(oxy))bis(2,1-phenylene))bis(benzo[d]thiazole) (**7g**)*: mp: 155-160°C; R_f: 0.70; HPLC purity (93.0%, eluent: 70% MeOH/ACN, Rt: 0.40 min); ^{1}H NMR (400 MHz, $CDCl_3$): σ 8.49 (d, *J* = 7.8 Hz, 2H), 8.07 (m, 3H), 7.68 (d, *J* = 8 Hz, 2H), 7.53-7.46 (m, 3H), 7.31 (t, *J* = 8.0, 4H), 7.18 (t, *J* = 8.0 Hz, 2H), 7.12-7.06 (m, 3H), 5.84 (d, *J* = 3.92 Hz, 4H), 4.02 (s, 3H); ^{13}C NMR ($CDCl_3$, 100 MHz); 60.4 (OMe), 71.1 (CH_2), 106.5, 113.0, 113.0, 121.3, 121.5, 121.7, 121.7, 122.2, 122.4, 122.4, 124.3, 124.6, 125.5, 125.9, 126.7, 129.8, 130.2, 131.7, 135.8, 137.6, 143.3, 147.6, 150.5, 152.1, 156.0, 161.6, 162.7, 171.1; IR (KBr, cm^{-1}): 2956.8, 1616.3, 1490.9, 1450.4, 1211.3, 1112.9, 1022,2, 752.2; LC-MS (MeOH) for $C_{37}H_{27}N_4O_3S_2$ $[M+H]^+$ Calcd:= 639.15, found: 639.3.

In vitro cytotoxic activities (MTT assay):

The basic detection of cytotoxicity of anticancer compounds *in vitro* is MTT assay. The MTT proliferation assay is based on the reduction of the yellow MTT tetrazolium salt (3-[4,5-dimethylthiazol-2-yl]-2,5diphenyltetrazolium bromide) by mitochondrial dehydrogenases to form a purple MTT formazan crystals in viable cells. Synthesized compounds (**7a-g**) were prior dissolved in 0.1% DMSO and then serial dilution with medium and used in the experiment. Three different types of cancer cell lines were used in this experiment i.e., human Epitheloid Cervix Carcinoma (HeLa), human colon cancer cell line (Caco-2) and one normal kidney cell (HEK-293) were used in the assay. The cells were harvested at the log phase and it is cell counted using haemocytometer. Approximately 1×10^3 cells per well were seeded using all the cell lines. They were cultured in 100 µL of a DMEM media in 96-well plates and incubated at 37 °C for 24 hours under a 5% CO_2 atmosphere. The cells were then washed with 1X PBS buffer and fresh media was added to the cells. The cells were then treated with different concentrations of the drugs (0.65-150 µM) in the volume of 100 µM /well. Cisplatin has been used as a standard positive control drug. The control wells contains the same cell count of 1×10^3 cells per well of 100 µL medium containing 0.1% DMSO. After 24 h (for HeLa cell and HEK-293) and 72 h (for Caco-2), the medium was discarded and it is washed with 1X PBS and it was incubated with 100 µl of MTT reagent (1 mg/ml) for 4 h at 37°C.

Then the 96 well plate was placed on the shaker for 10 min and subsequently the absorbance was recorded by the ELISA reader at λ = 490 nm. The experiments were performed in triplicate. The data were expressed as the growth inhibition percentage calculated according to the equation: % growth inhibition = 100 − [(AD × 100)/AB], where AD is the absorbance measured for sample and AB is the absorbance measured for blank wells (cells with a medium and a vehicle).

2.5. Cellular imaging assay

HeLa cell line was procured from NCCS and used for this study. Cellular uptake studies of the drug were carried out in 24 well plates. The cells were cultured, when it attained 80% confluency it was taken for the bio imaging studies. The cells were trypsinized using 1-2ml of 1X trypsin. After detachment of cells, 2ml of DMEM media was added. Then it was transferred to fresh 15 ml falcon tube and centrifuged for 2000 rpm for 1-5 min. DMEM fresh media (80µl) was added to the pellet formed at the bottom of the tube and the cells were seeded in 24 well plates. Then Sample drug in PBS buffer was added to well plates. After incubating for 4 h at 37 °C, all the cells were washed with PBS buffer (pH 7.4). Then, the fluorescence images were recorded with an Olympus Fluorescence microscope with 480-550 nm excitations.

2.6. DNA binding experiment

Interaction of complex (**7e**) with DNA was recorded using UV-Visible absorption spectroscopy and ethidium bromide displacement assay by fluorescence spectroscopy.

2.6.1. UV-visible spectral studies

The DNA binding assay were carried out using Tris-HCl as a buffer (5 mM L^{-1} Tris-HCl /50mM L^{-1} NaCl in water, pH 7.4) using an aqueous solution of the compound **7e**. The purity of the CT-DNA was checked using nanodrop instrument and it was found to be 1.8:1 which indicated that DNA are free from proteins. The CT-DNA concentration in buffer were calculated by using the UV absorbance value at 260 nm and from the known molar absorption coefficient value and it was found to be 6600 M^{-1} cm^{-1} [1]. DNA binding experiments were performed by incubating constant complex concentration and increasing CT-DNA concentration [for 5 minute at room temperature] to give sufficient time to interact

with DNA. The absorbance of the complex was measured after each succeeding addition of CT-DNA.

The intrinsic DNA binding constant (K_b) was obtained using the equation (1)

$$\frac{[DNA]}{(\varepsilon_a - \varepsilon_f)} = \frac{[DNA]}{(\varepsilon_b - \varepsilon_f)} + \frac{1}{K_b(\varepsilon_a - \varepsilon_f)} (1)$$

Where [DNA] is the concentration of DNA in the base pairs, $\boldsymbol{\varepsilon}_a$ is the apparent extinction coefficient, $\boldsymbol{\varepsilon}_f$ is the extinction coefficient of the complex in its free form, and $\boldsymbol{\varepsilon}_b$ refers to the extinction coefficient of the complex when fully bound to DNA. Data were plotted using Origin Lab, version 8.5 to obtain the [DNA]/($\boldsymbol{\varepsilon}_a$-$\boldsymbol{\varepsilon}_f$) vs. [DNA] linear plots. The ratio of the slope to intercept from the linear fit gives the value of the intrinsic binding constant (K_b).

2.6.2. Fluorescence study:

To know the emission property of all the synthesized complexes (**7a-g**), an initial investigation was performed using spectrofluorometric method in water by using absorption maximum as their excitation energy. Fluorescence quantum yield (Φ) of all the prepared compounds performed in water solution were calculated by employing the comparative William's method which involves the use of well-characterized standard with the known quantum yield value. Quinine sulphate was used as reference fluorophore excited at 350 nm and emission at 452 nm, quantum yield (Φ_R) = 0.50 in 1N H_2SO_4. The gradients of the plots are proportional to the quantum yield (Φ) of the studied system. The data obtained and quantum yield value calculated according to the equation (2):

$$\Phi = \Phi_R \times (I_S / I_R) \times (OD_R / OD_S) \times (\eta_S / \eta_R)(2)$$

Where, φ = Quantum yield, I = Peak Area, OD = absorbance at $\lambda_{max,}$ η = Refractive index of solvent and reference. Here, we have used Quinine Sulphate as a standard for calculating emission of quantum yield. 0.5 M H_2SO_4 was used as solvent for the standard and water for synthesized compounds.

DNA binding titration was also carried out in Tris-HCl buffer (5 mM Tris-HCl in water, pH 7.4) using aqueous solution of complex **7e** by fluorescence spectroscopic method. Following successive addition of CT-DNA, the sample was kept for 5 min. After completion of this titration, the graph was plotted between Intensity and Wavelength.

2.6.3. Ethidium bromide displacement assay

EtBr is also known as a trypanocidal dye which has numerous biological applications [2, 3]. EtBr displacement is periodically used as a diagnostic approach to determine the capability of compounds to bind with DNA [4]. EtBr emits intense fluorescence when it intercalates with CT-DNA, whereas in its free form in buffer (5 mM Tris-HCl/50mM NaCl buffer, pH 7.4) there is no apparent florescence emission. Introduction of the second molecule that has efficiency to bind with DNA it replaces the EtBr bounded to CT-DNA or it diminish the excited state of EtBr-CT-DNA by means of molecular collision [5]. The binding capacity of drug complex with CT-DNA was estimated by quenching of fluorescence intensity. The apparent binding constant (K_{app}) of the complex to CT DNA were calculating by plotting fluorescent intensity versus complex concentration by using the following equation:

$$\mathrm{K}_{app} \times [Complex]_{50} = \mathrm{K}_{EtBr} \times [EtBr] (3)$$

where K_{app} is the apparent binding constant of the complex, $[Complex]_{50}$ is the concentration of the complex at 50% fluorescence quenching of DNA-bound ethidium bromide, K_{EB} is the binding constant of the EB ($K_{EtBr} = 1.0 \times 10^7\ M^{-1}$), and [EB] is the concentration of ethidium bromide (8 μM). The Stern-Volmer quenching constant (K_{SV}) has been determined quantitatively by using Stern-Volmer equation. Stern-Volmer plots of I_0/I vs. [complex] were made using the corrected fluorescence data taking into account the effect of dilution. Linear fit of the data using the equation:

$$I_0/I = 1 + \mathrm{K}_{SV}\ [Q] (4)$$

Where I_0 and I are the emission intensities of EB- CTDNA in the absence and in the presence of complex of concentration [Q], gave the quenching constant (K_{SV}) using Origin Pro 8.5 software.

2.7. BSA binding study

BSA is a carrier protein that increases solubility of the drug in the blood by binding to it [6]. Fluorescence spectroscopy is an efficient method to study the interaction of the BSA with the compound by quenching mechanism [7]. The binding of the complexes to the bovine serum albumin (BSA) were measured by tryptophan emission quenching study. The complex

solutions (0-60 μM) were gradually added to the solution of BSA (2 μM) in 5 mM Tris-HCl/NaCl buffer (pH 7.2) and the quenching of the emission signals at 350 nm (λ_{ex} = 295 nm) were recorded. The emission intensity of BSA decreases gradually on increasing the complex concentration. The quenching constant (K_{BSA}) has been determined quantitatively by using Stern-Volmer equation. Stern-Volmer plots of I_0/I vs. [complex] were made using the corrected fluorescence data taking into account the effect of dilution. Linear fit of the data using the equation:

$$I_0/I = 1 + \mathrm{K}_{BSA}[Q] = 1 + k_q\tau_0[Q] (5)$$

Where I_0 and I are the emission intensities of BSA in the absence and in the presence of quencher of concentration [Q], gave the quenching constant (K_{BSA}) using Origin Pro 8.5 software. k_q is the quenching rate constant, τ_0 is the average lifetime of the tryptophan in BSA without quencher reported as 1 x 10^{-8} sec. The binding propensity of the quenchers with respective serum proteins were expressed by Scatchard equation. For such static quenching interaction, the binding constant (K) and the number of binding sites (n) can be determined according to the Scatchard equation:

$$\log(I_0 - I/I) = \log K + n\log[Q] (6)$$

The linear fitting of the log $(I_0\text{-}I)/I$ vs. log [Q] plot gives values of n and K from slope and intercept.

2.8. Stability study, Solubility study, metabolic stability study, Caco-2 permeability study

$$\%\text{Nominal} = \frac{\text{Mean of calculated concentration of test samples}}{\text{Calculated concentration from calibration curve}} \times 100 \; \ldots.(7)$$

$$CL_{\text{intr}} = k \times \frac{\text{volume of reaction mixture (mL)}}{\text{protein content (mg)}} \; \ldots\ldots\ldots\ldots\ldots\ldots\ldots\ldots(8)$$

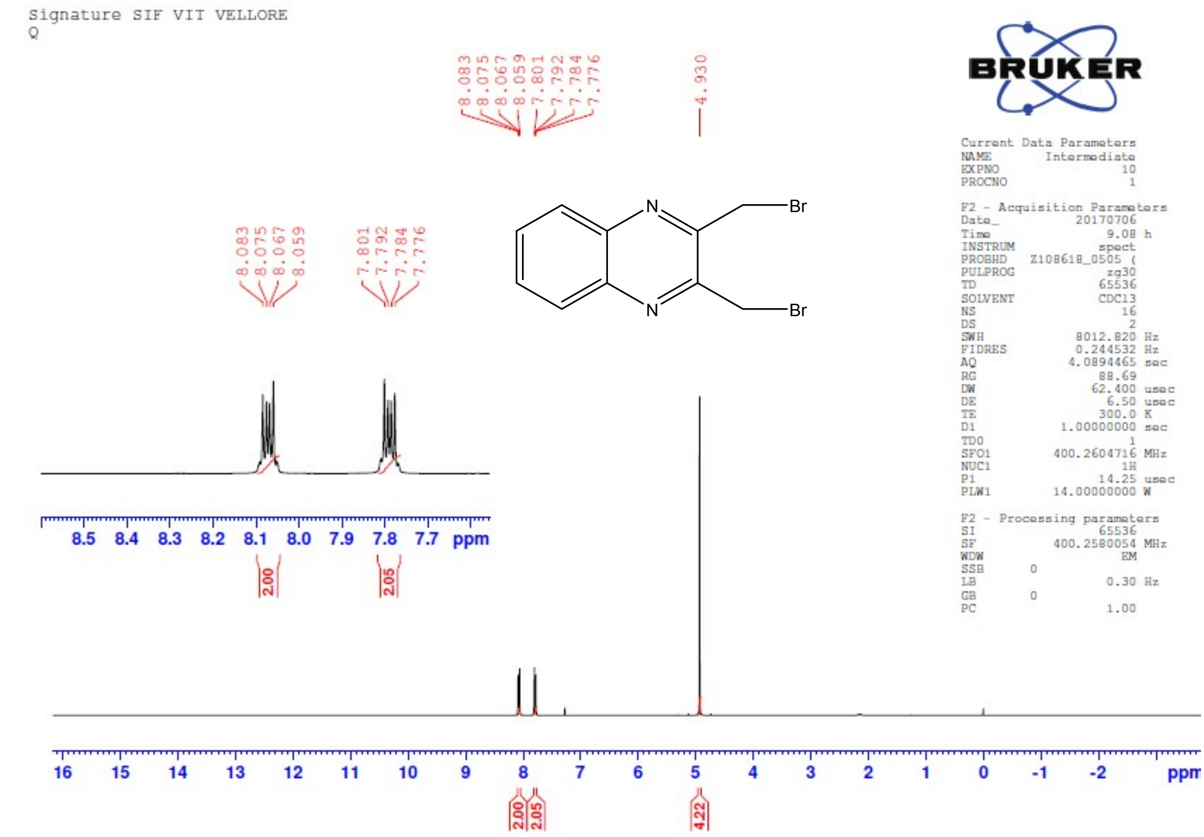

^{1}H NMR of compound **6a**

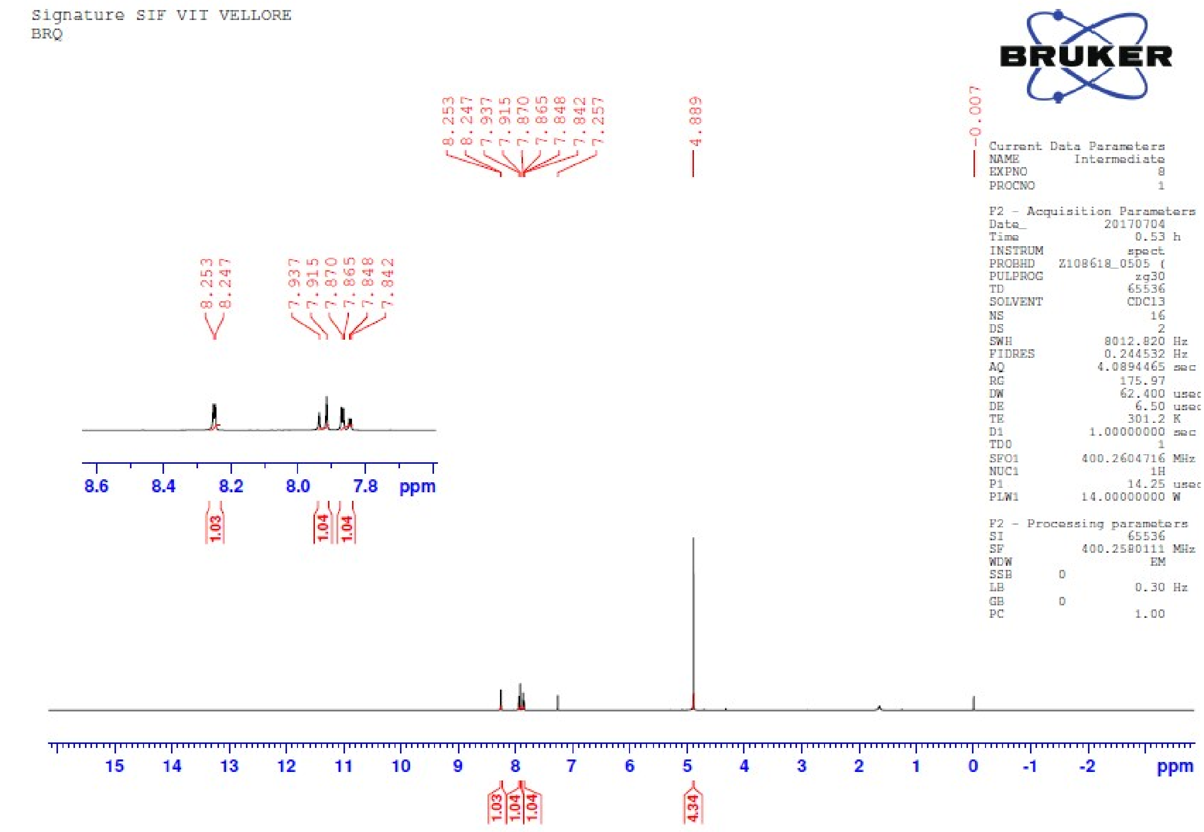

^{1}H NMR of compound **6b**

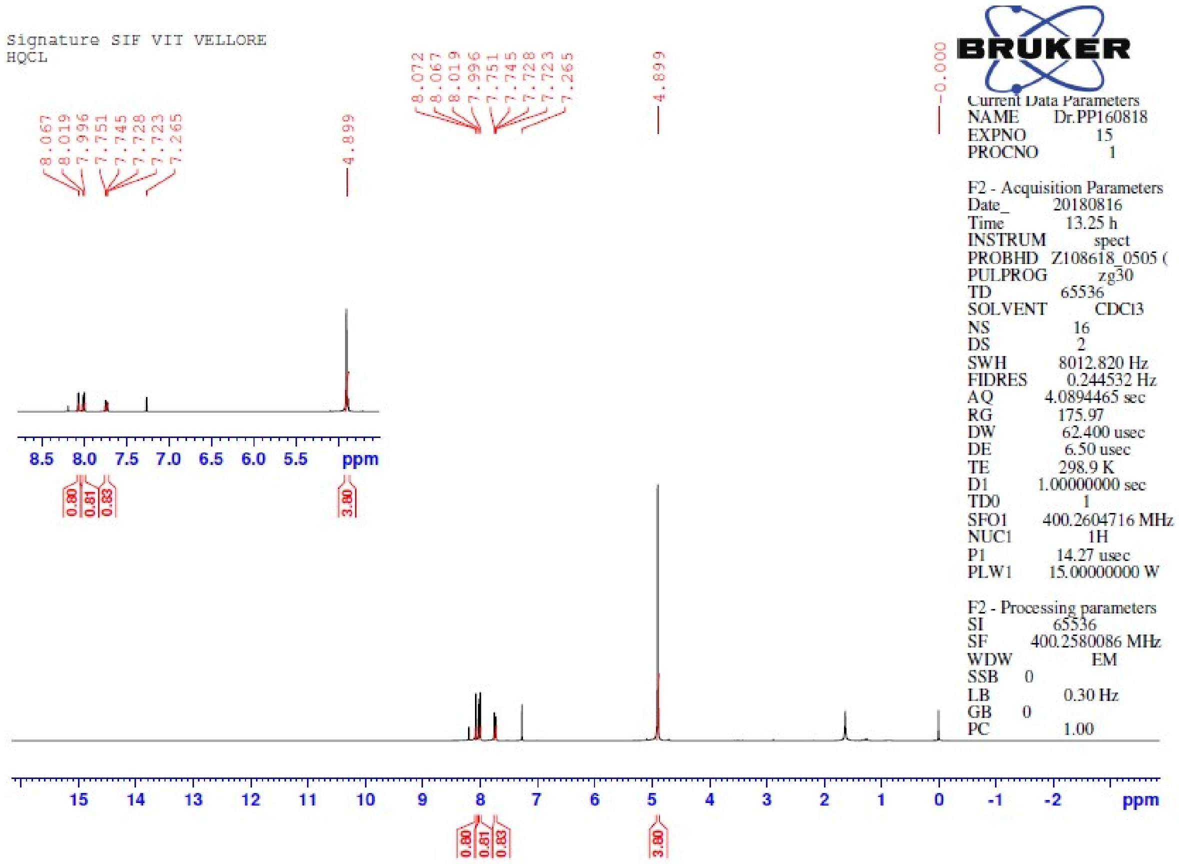

^{1}H NMR of compound **6c**

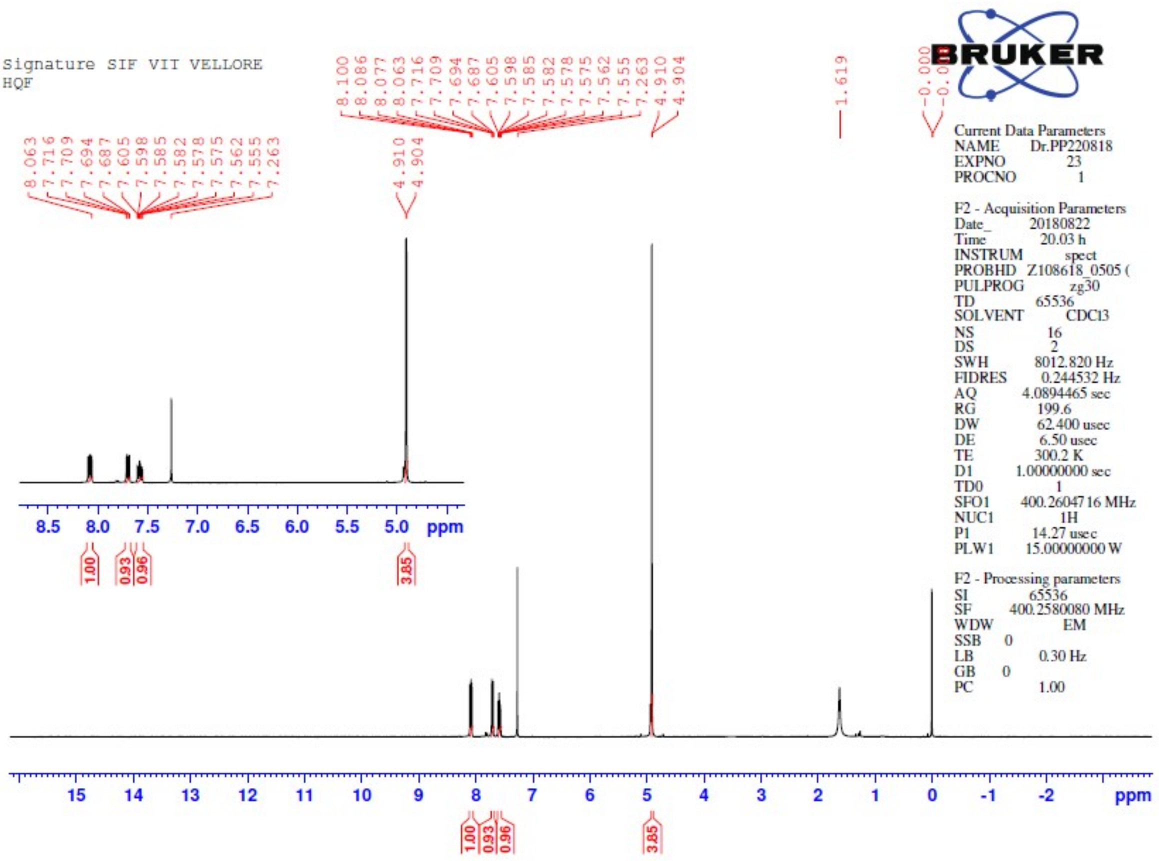

^{1}H NMR of compound **6d**

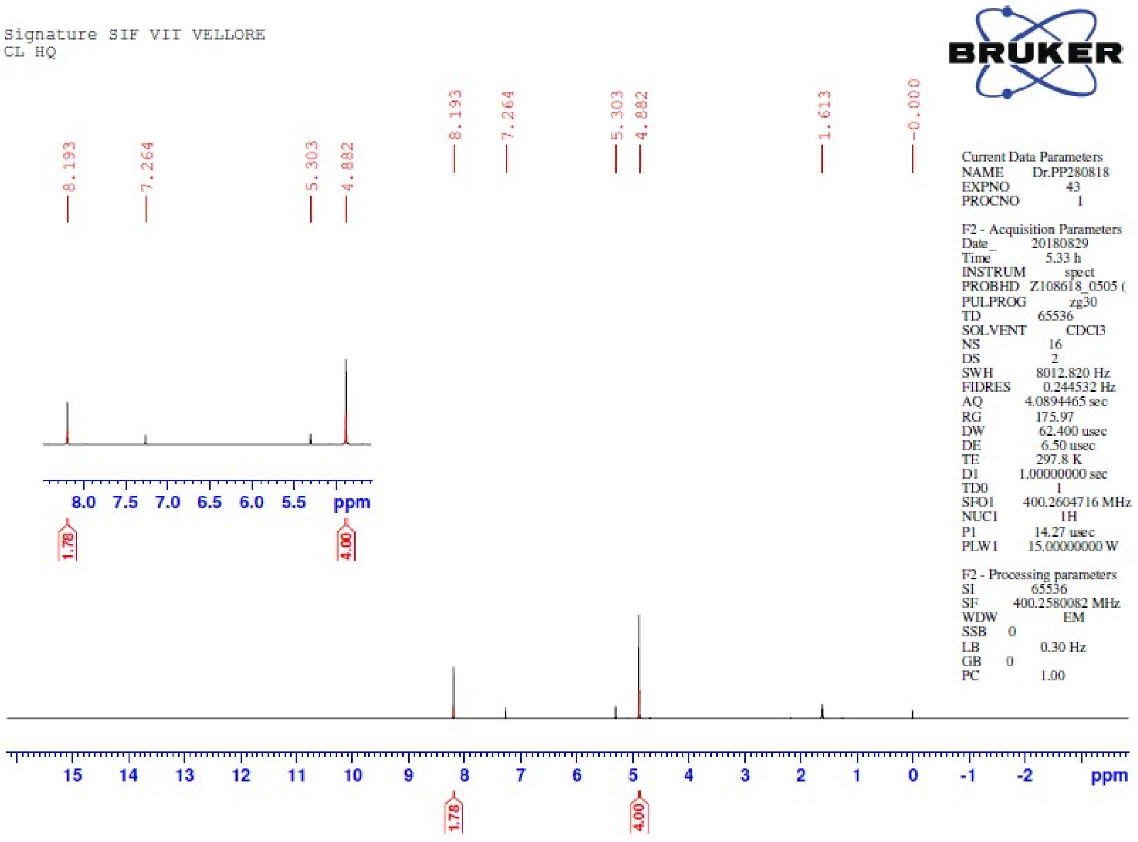

^{1}H NMR of compound **6e**

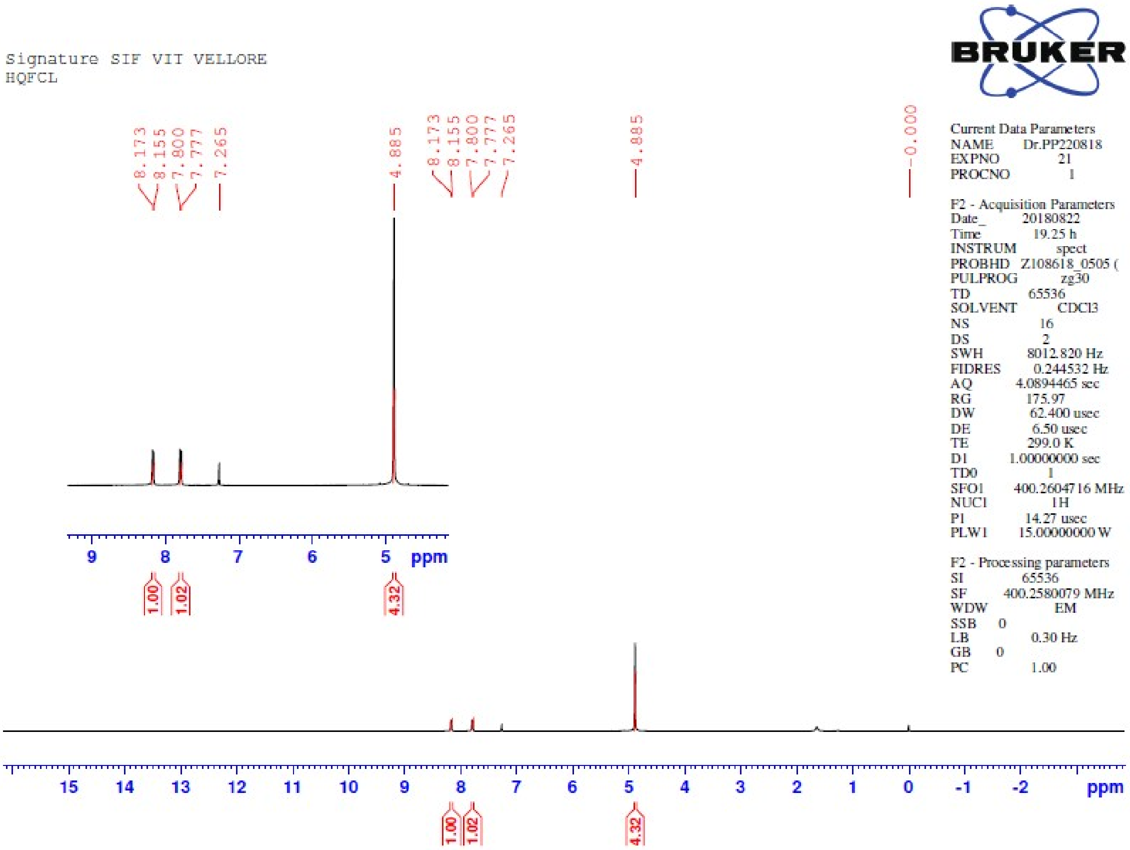

^{1}H NMR of compound **6f**

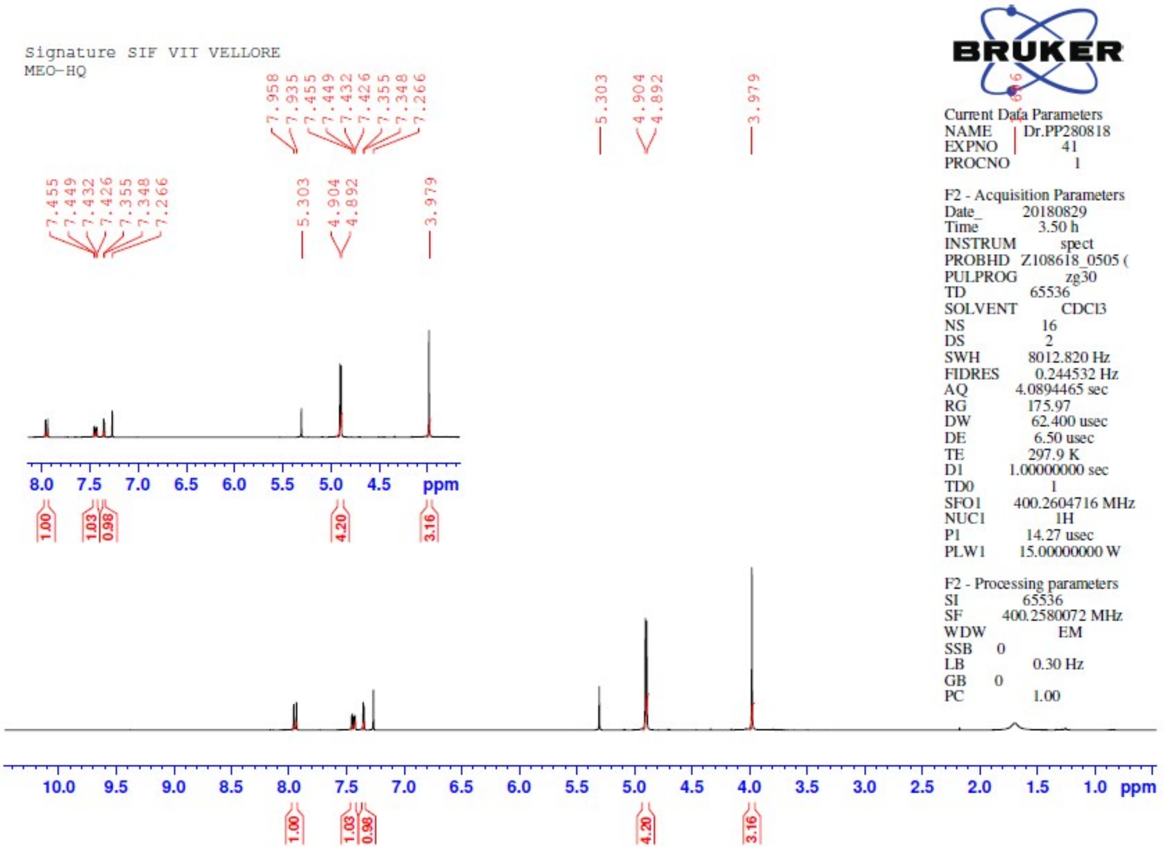

^{1}H NMR of compound **6g**

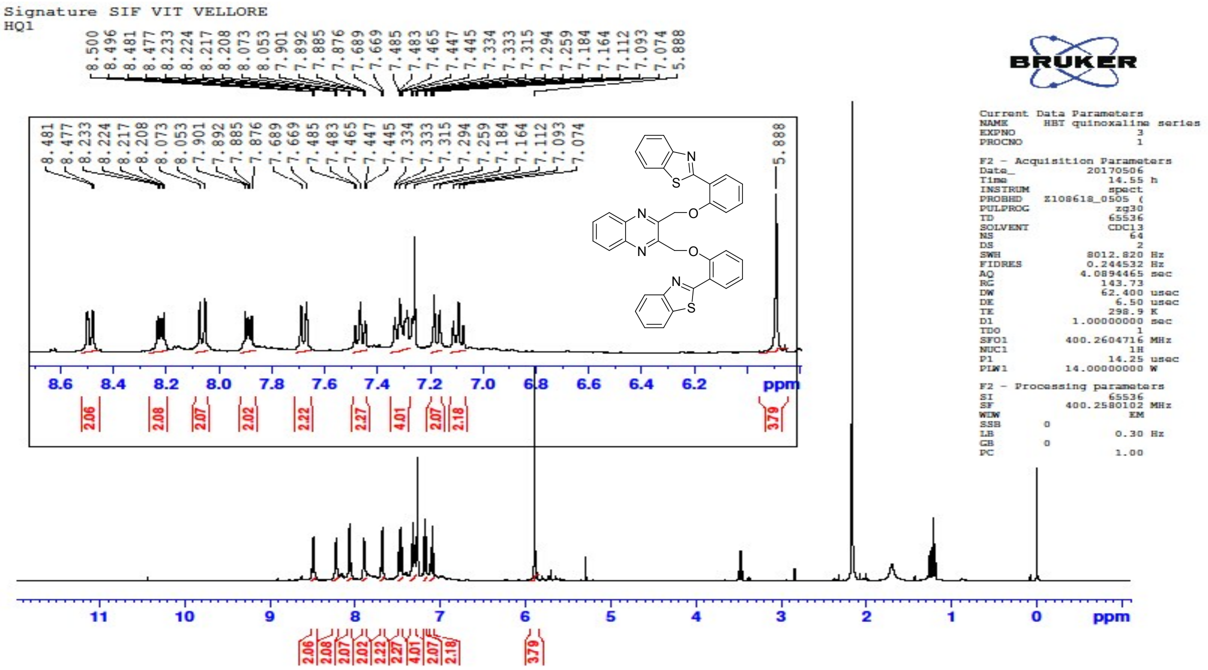

^{1}H NMR of compound **7a**

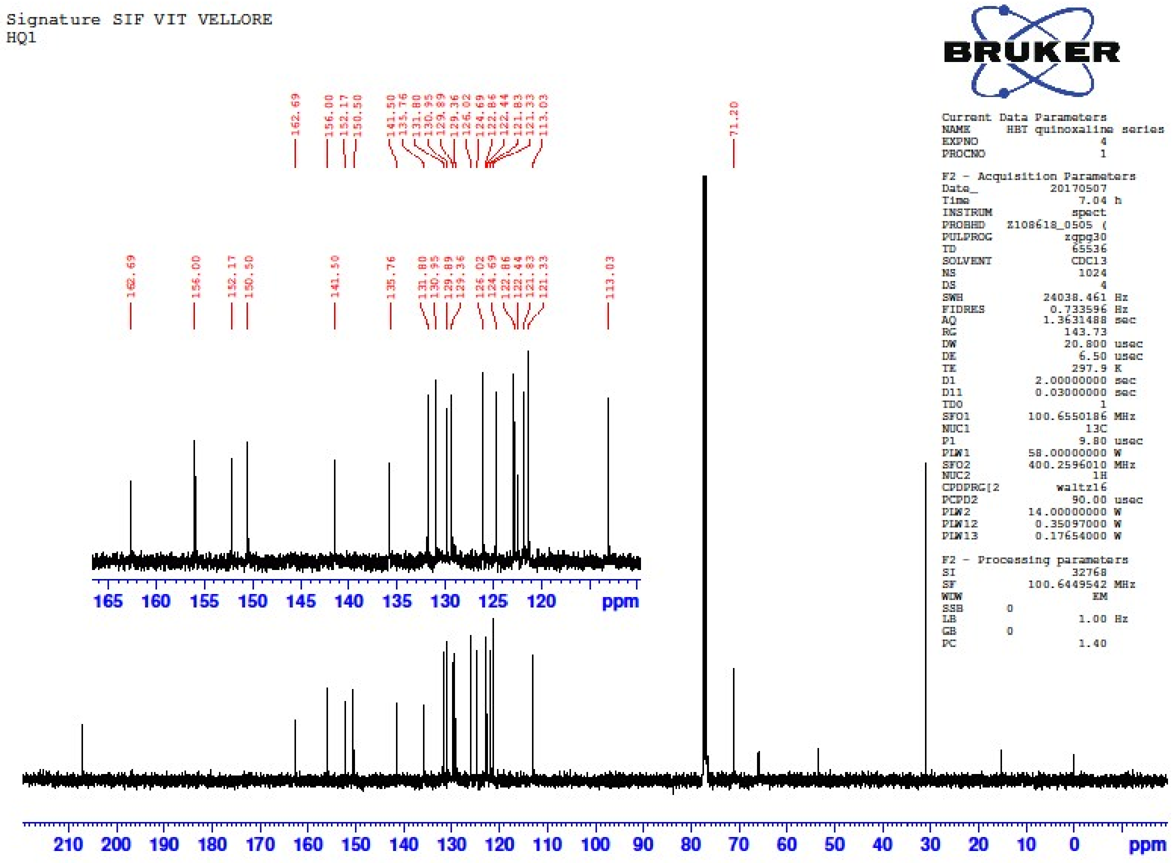

^{13}C NMR of compound **7a**

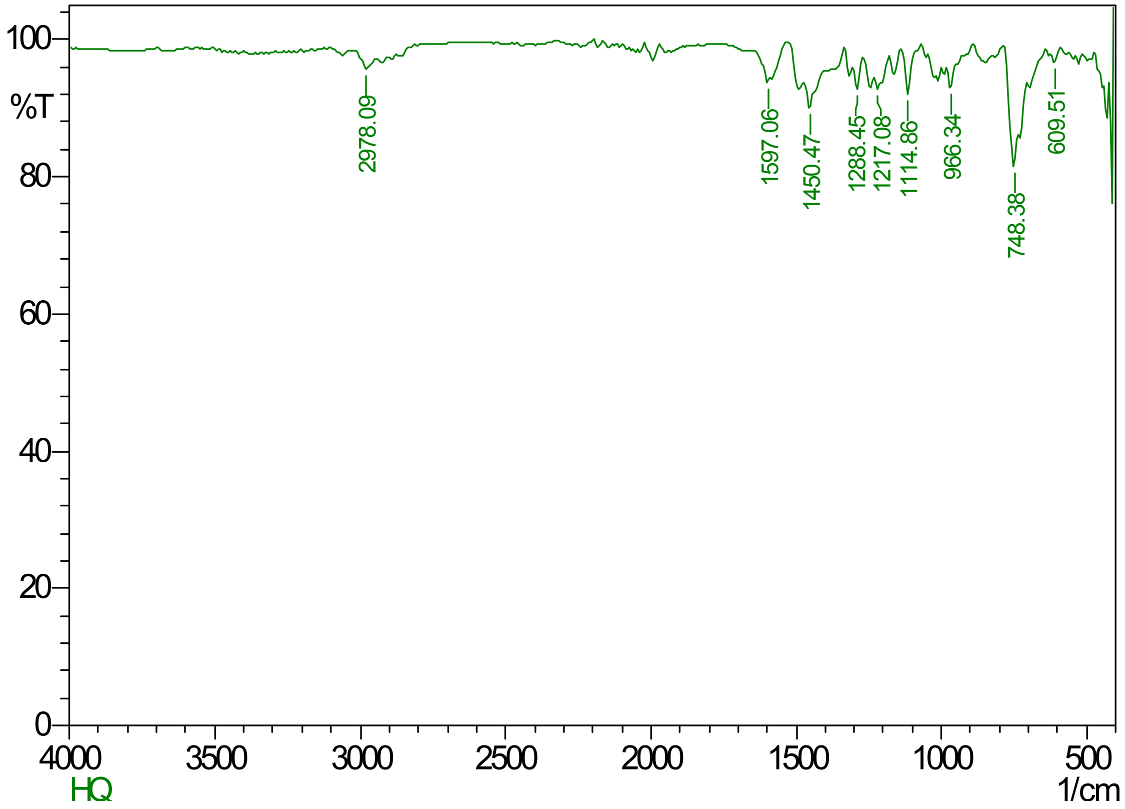

IR spectra of compound **7a**

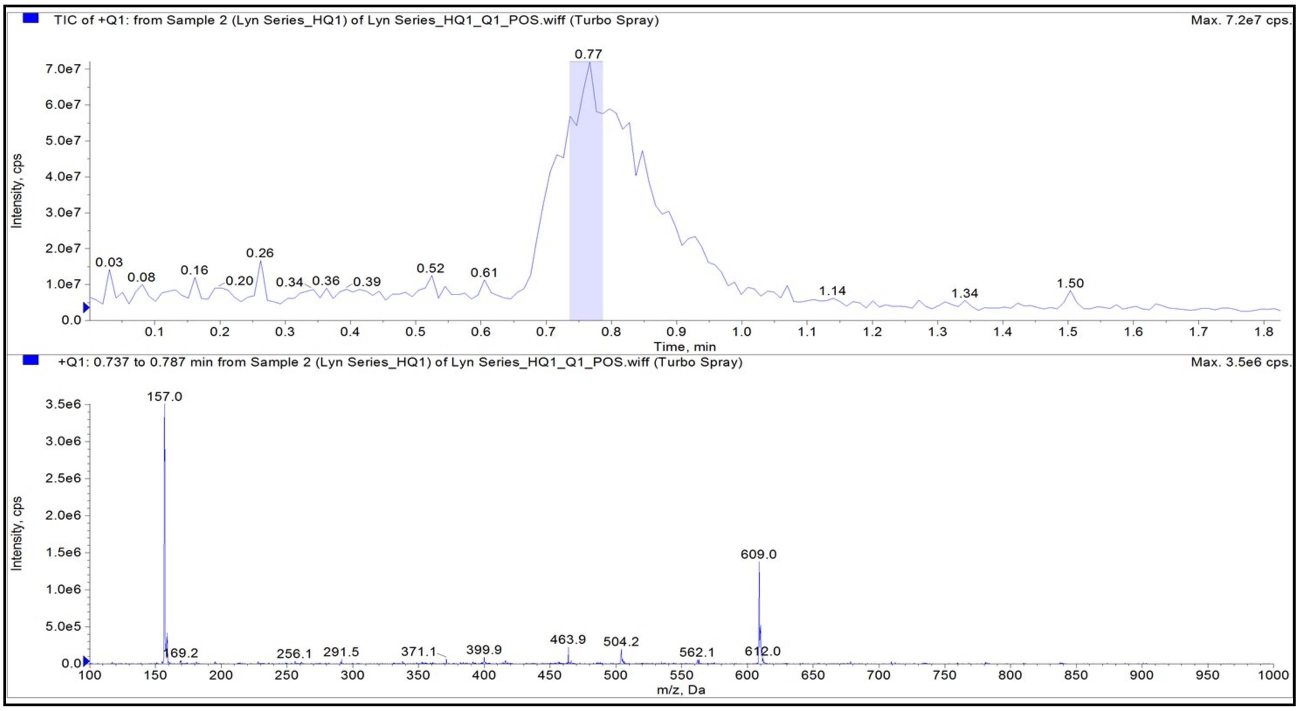

LCMS of compound **7a**

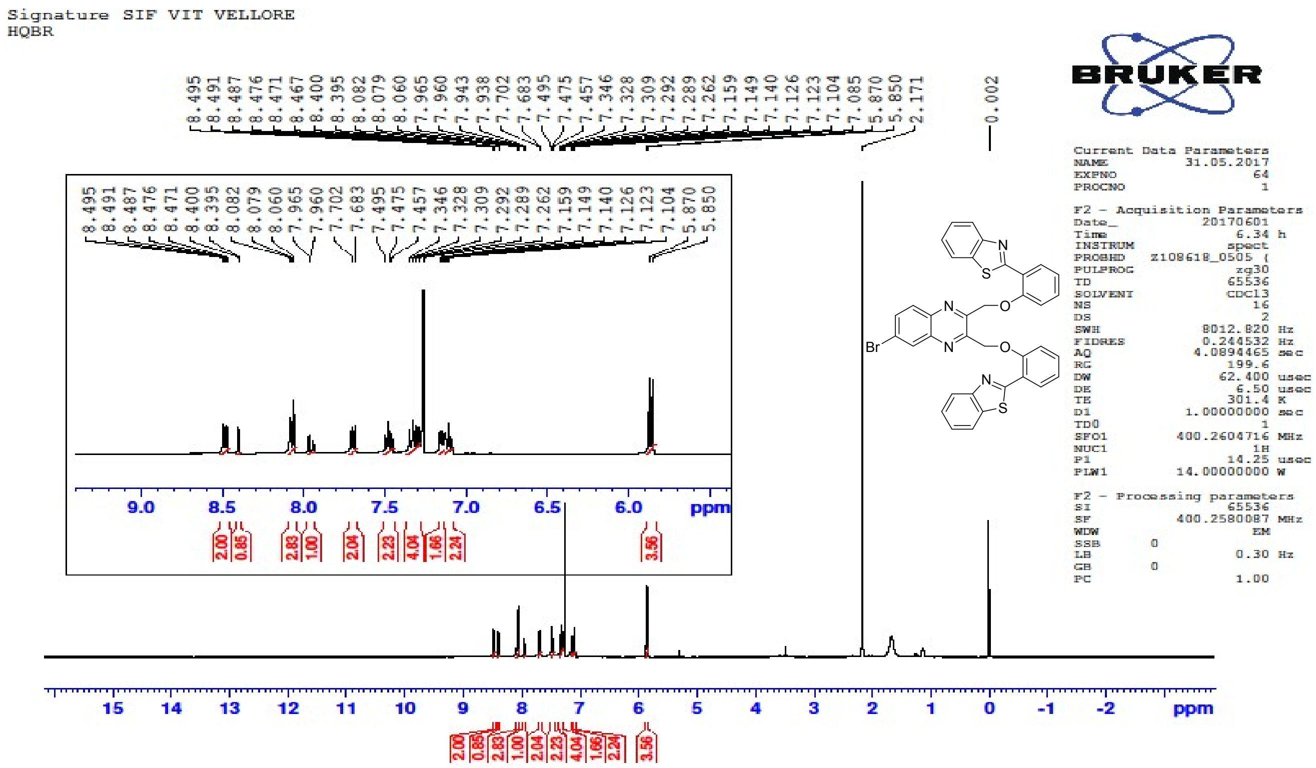

^{1}H NMR of compound **7b**

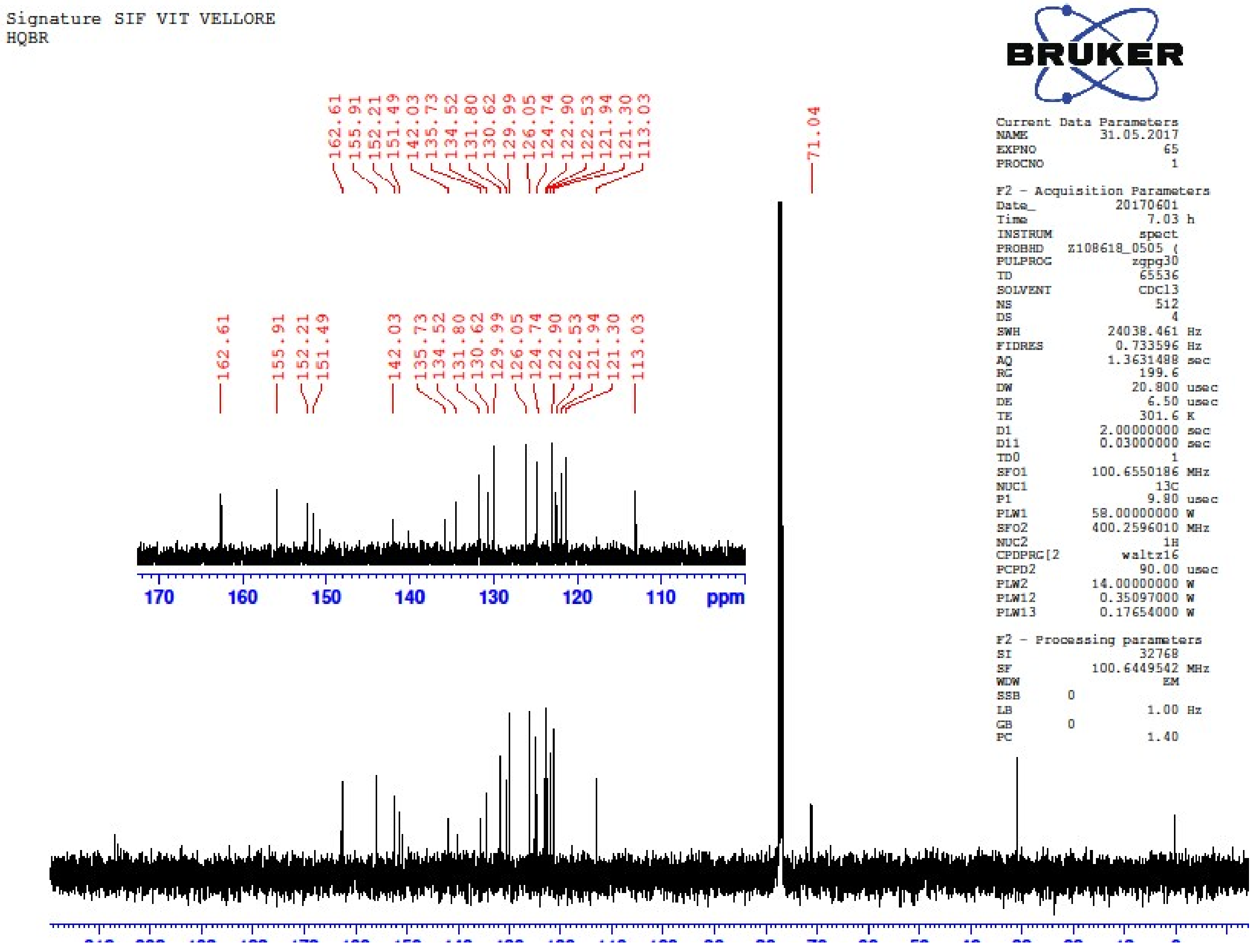

^{13}C NMR of compound **7b**

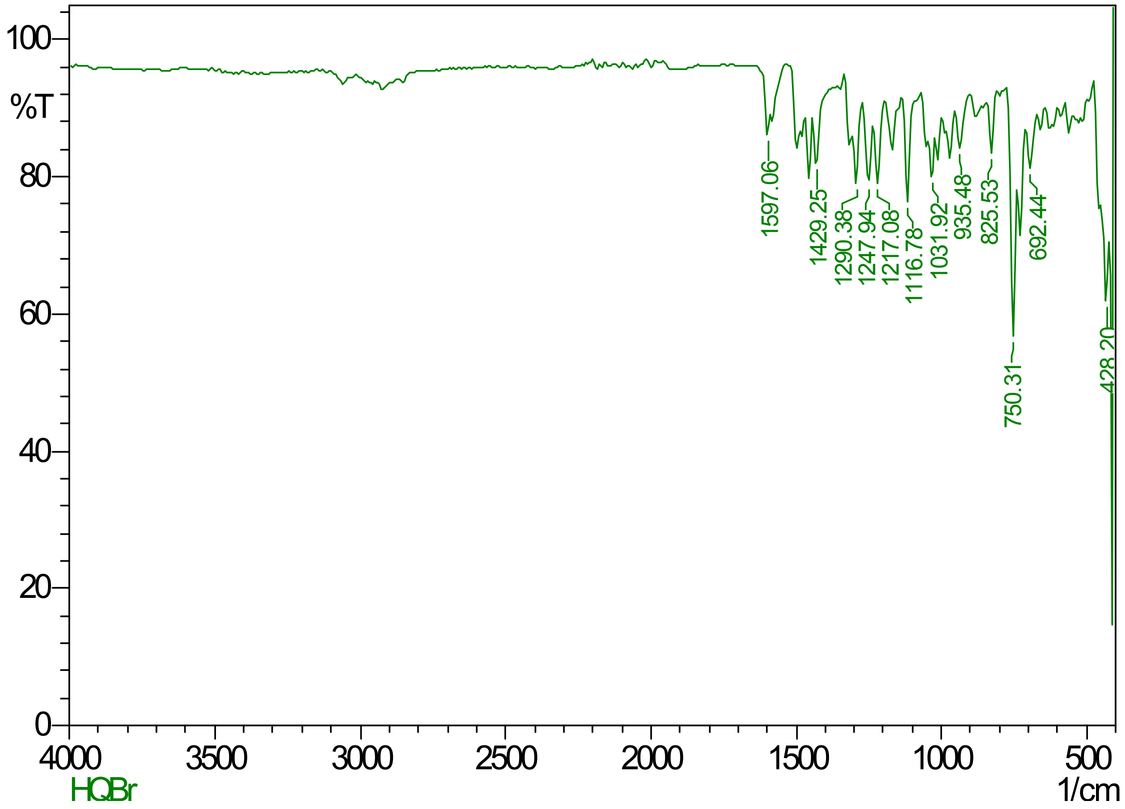

IR spectra of compound **7b**

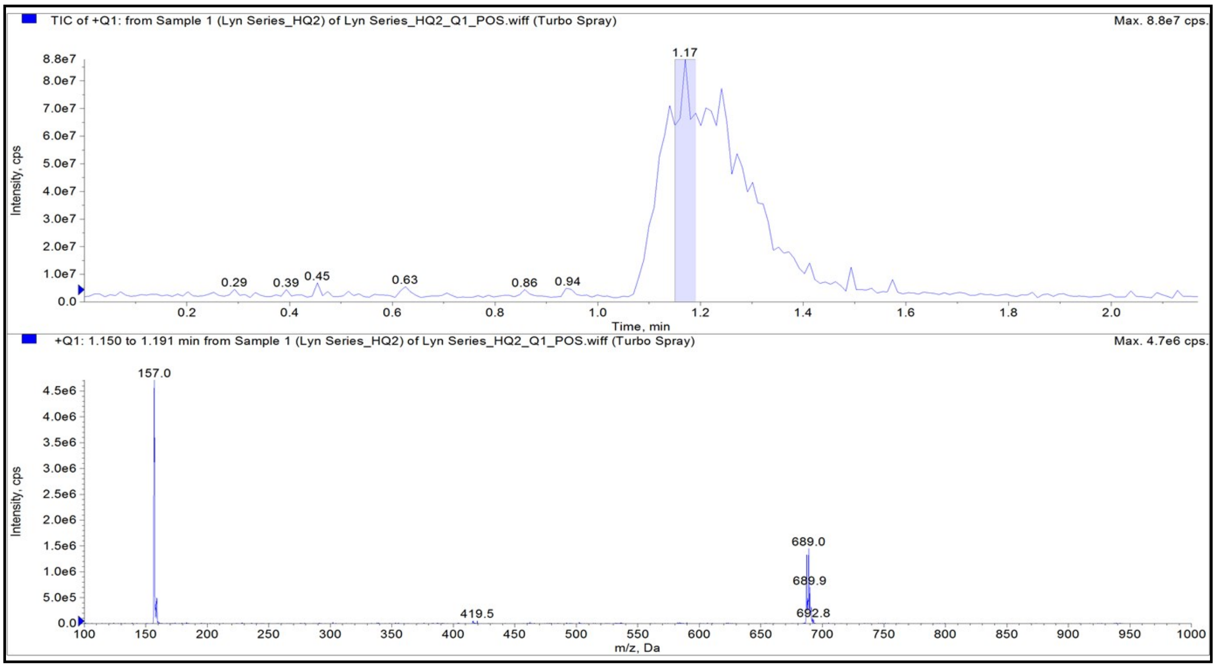

LCMS of compound **7b**

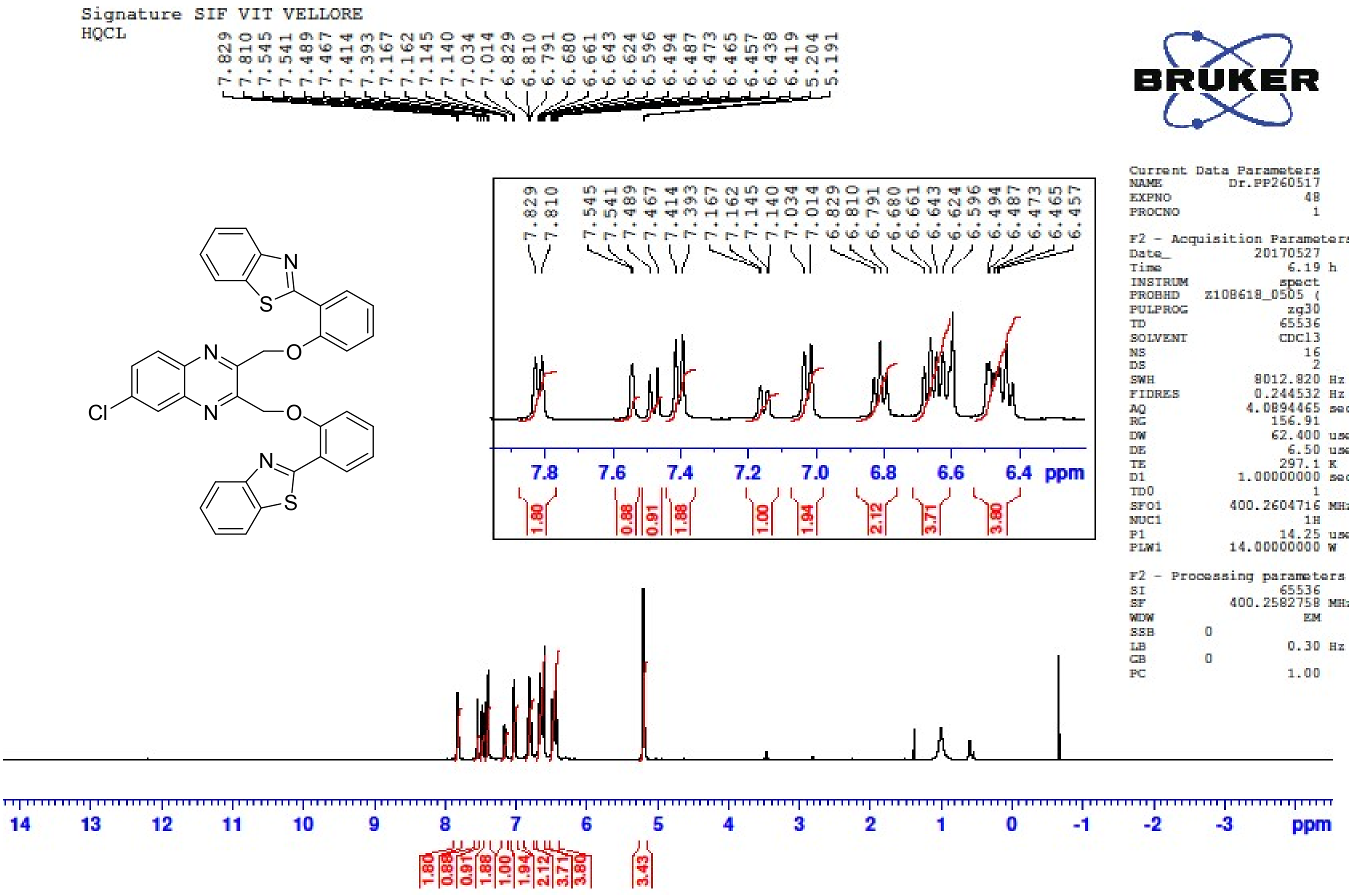

^{1}H NMR of compound **7c**

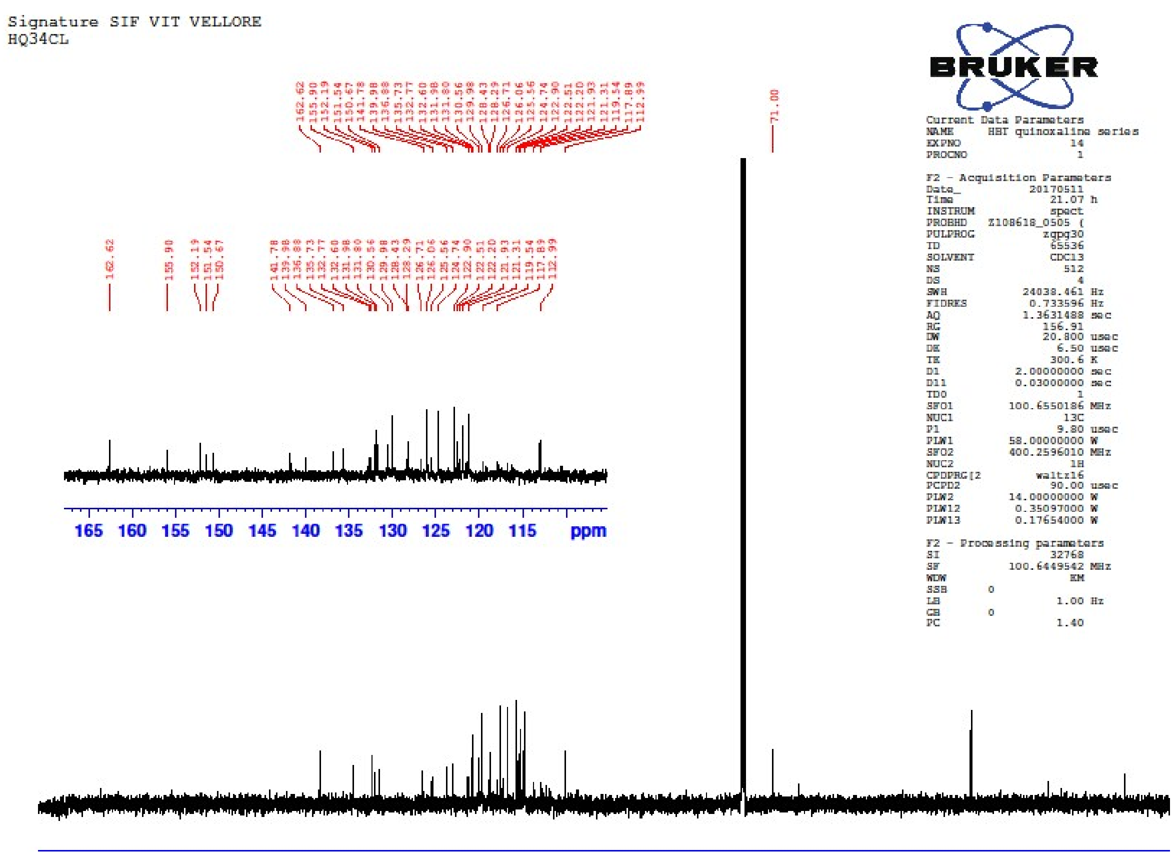

^{13}C NMR of compound **7c**

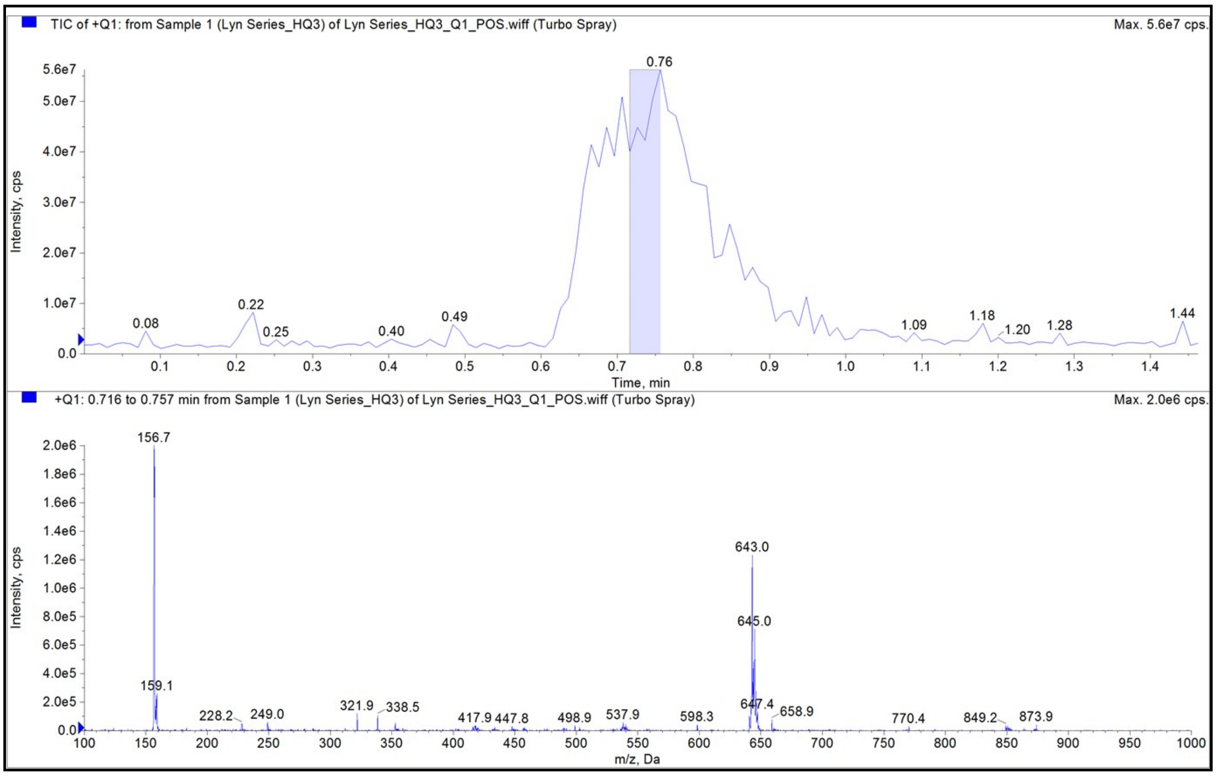

LCMS od compound **7c**

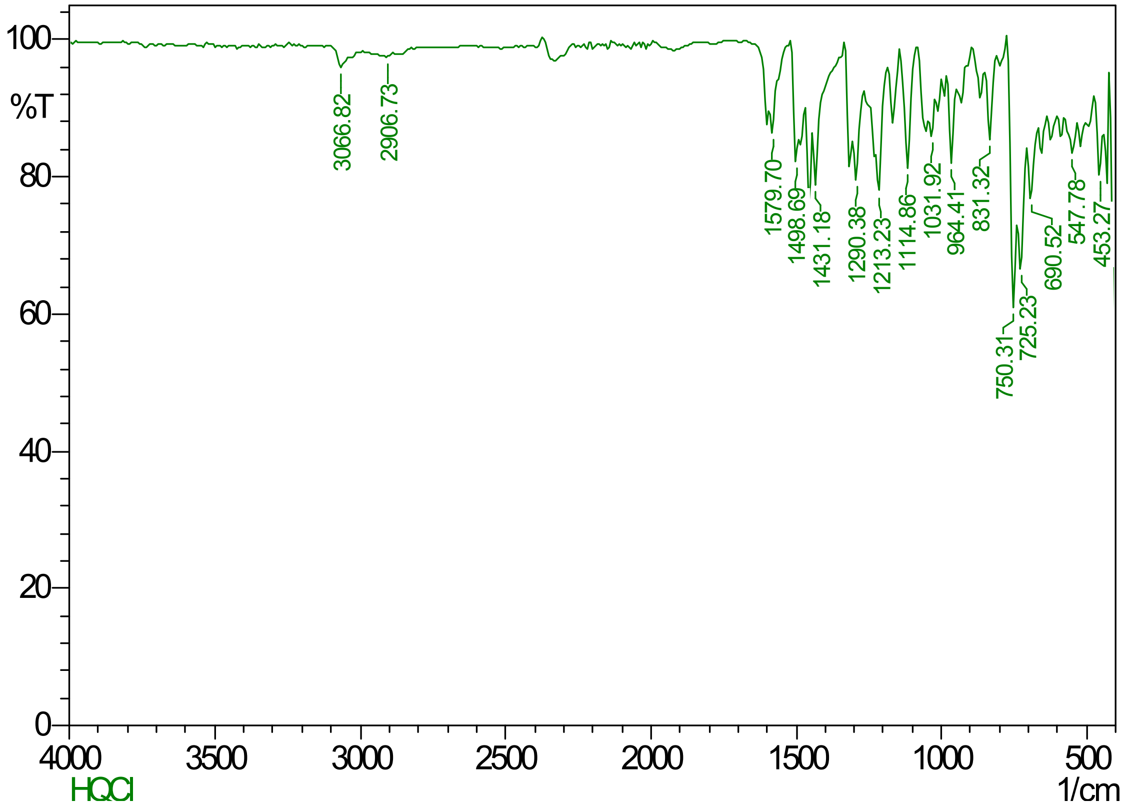

IR spectra of compound **7c**

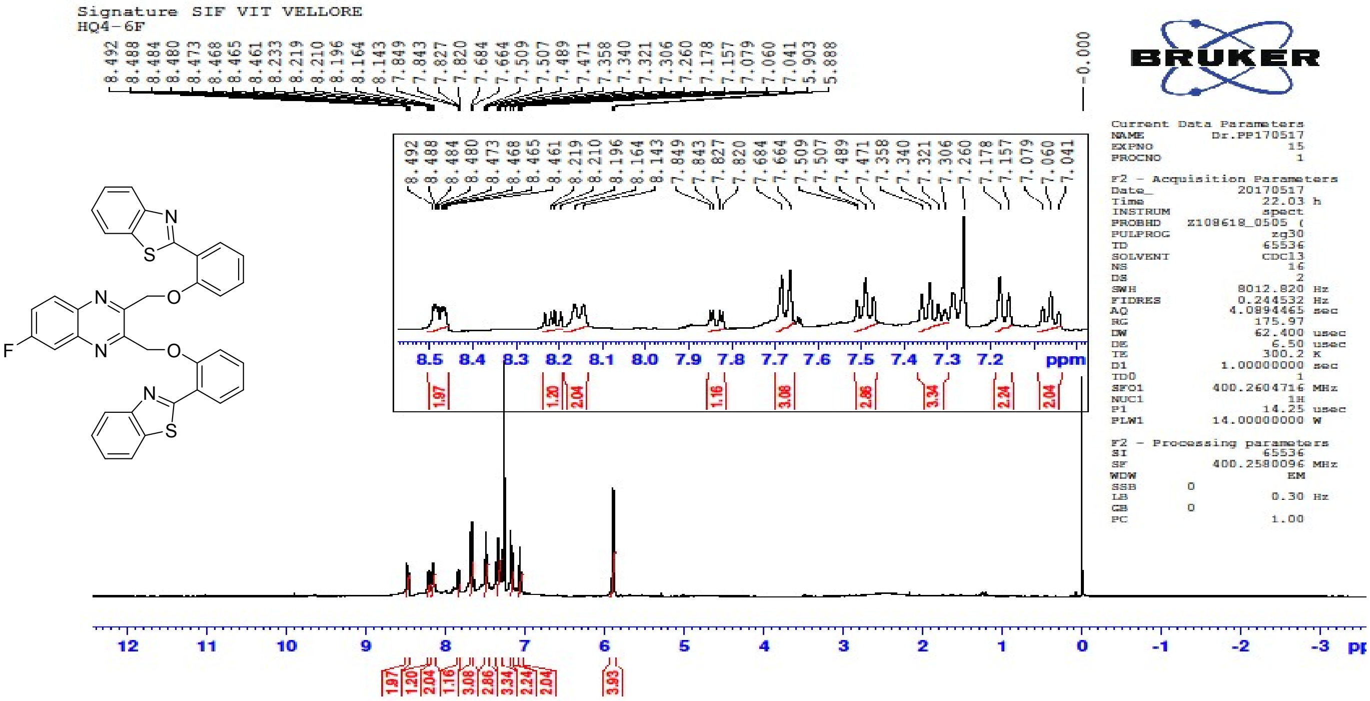

^{1}H NMR of compound **7d**

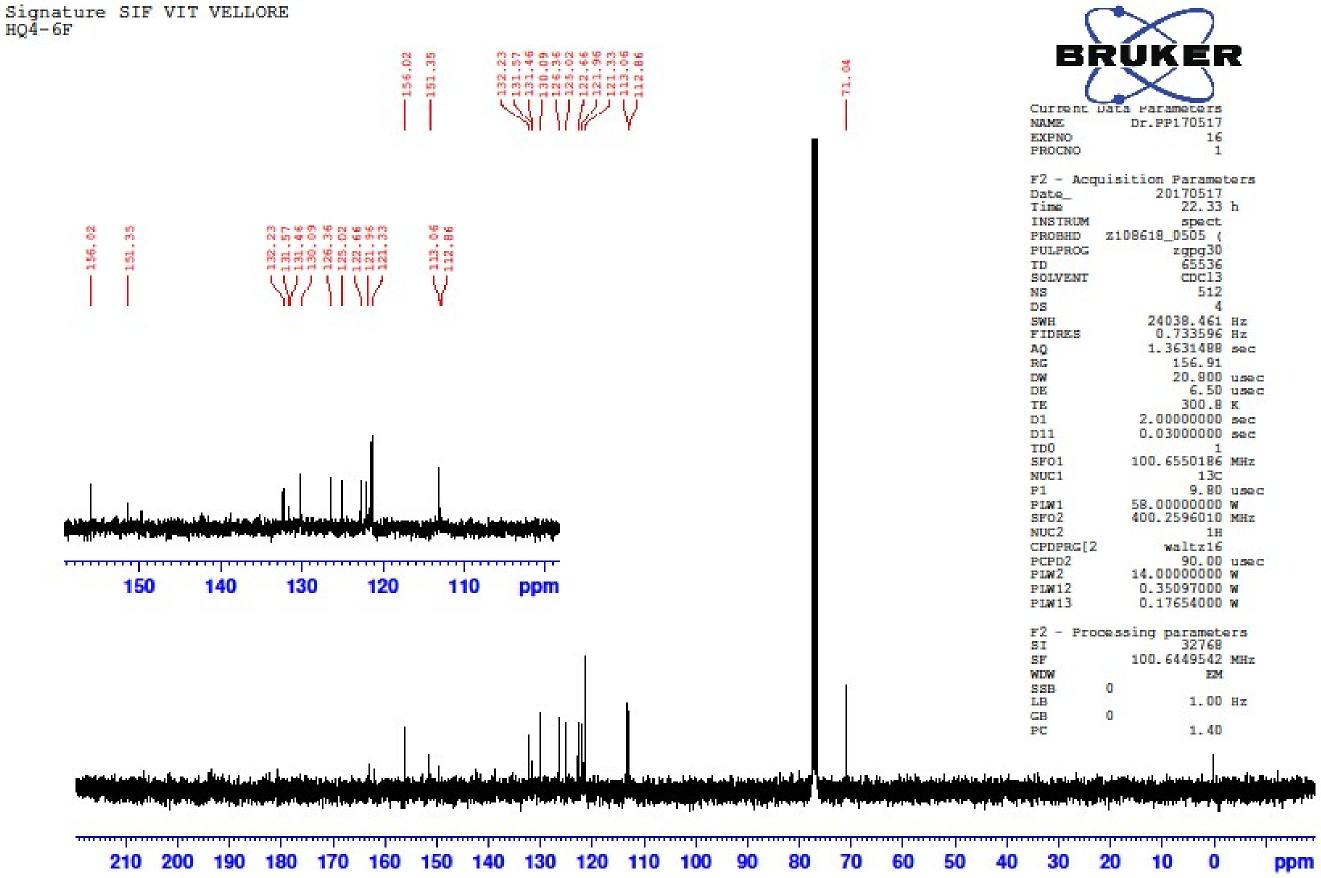

^{13}C NMR of compound **7d**

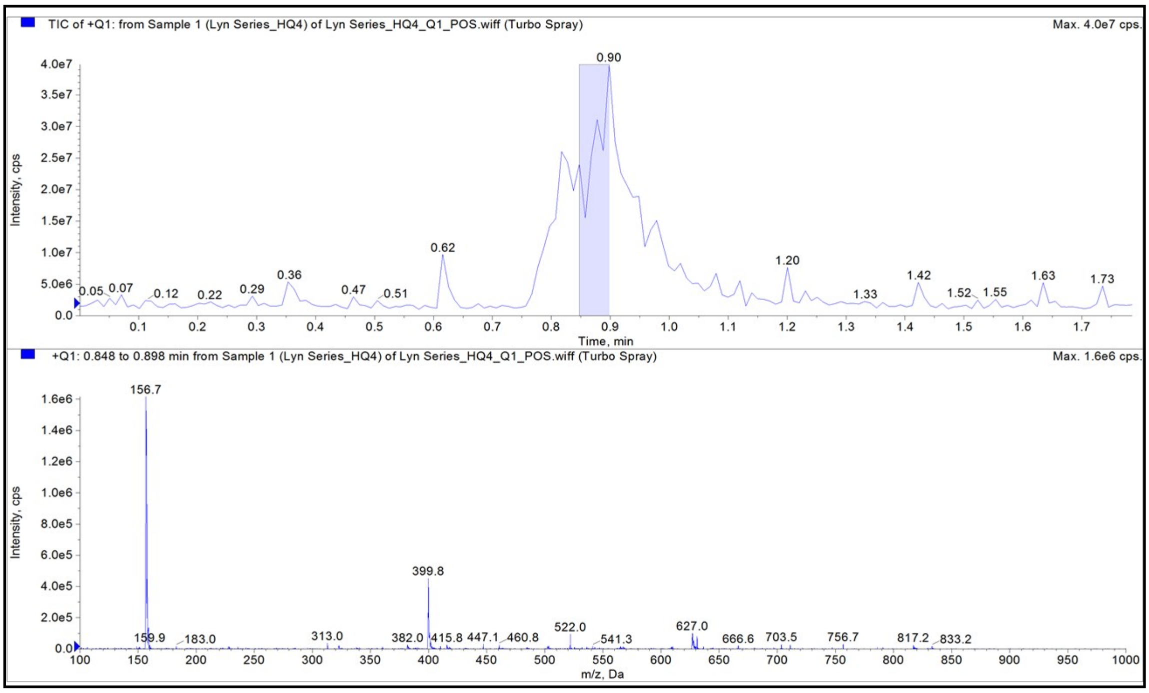

LCMS of compound **7d**

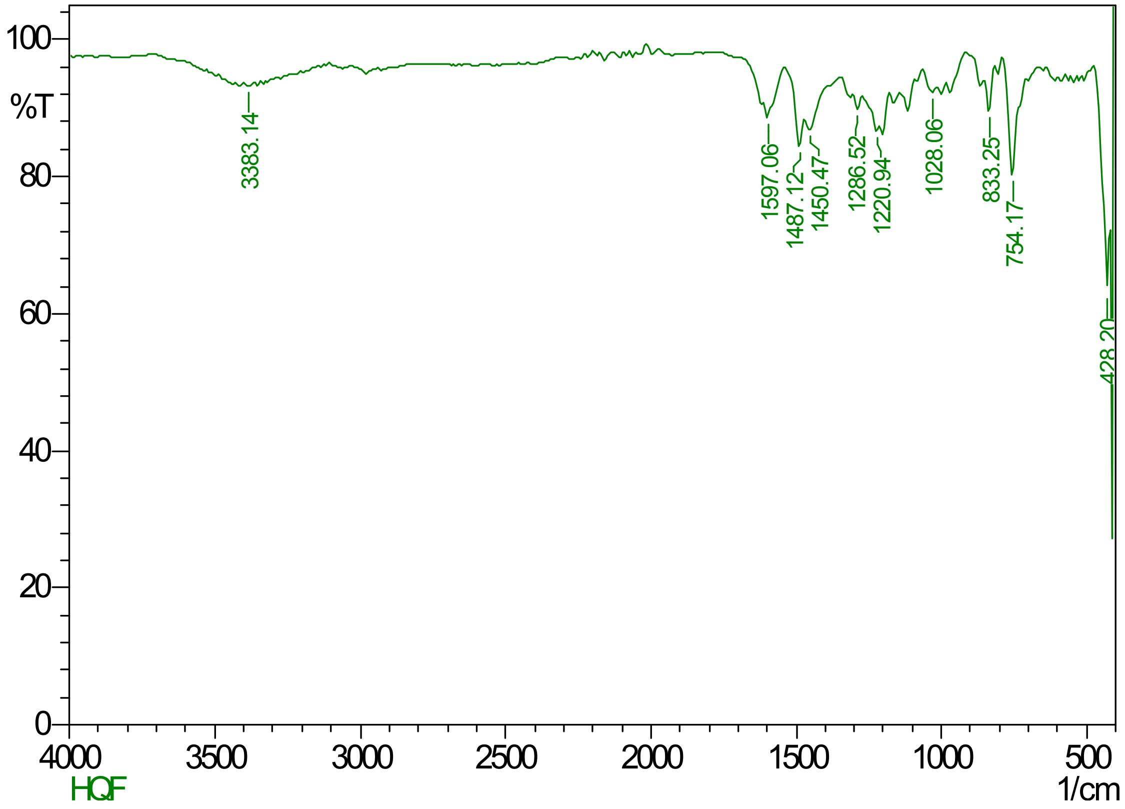

IR of compound **7d**

^{1}H NMR of compound **7e**

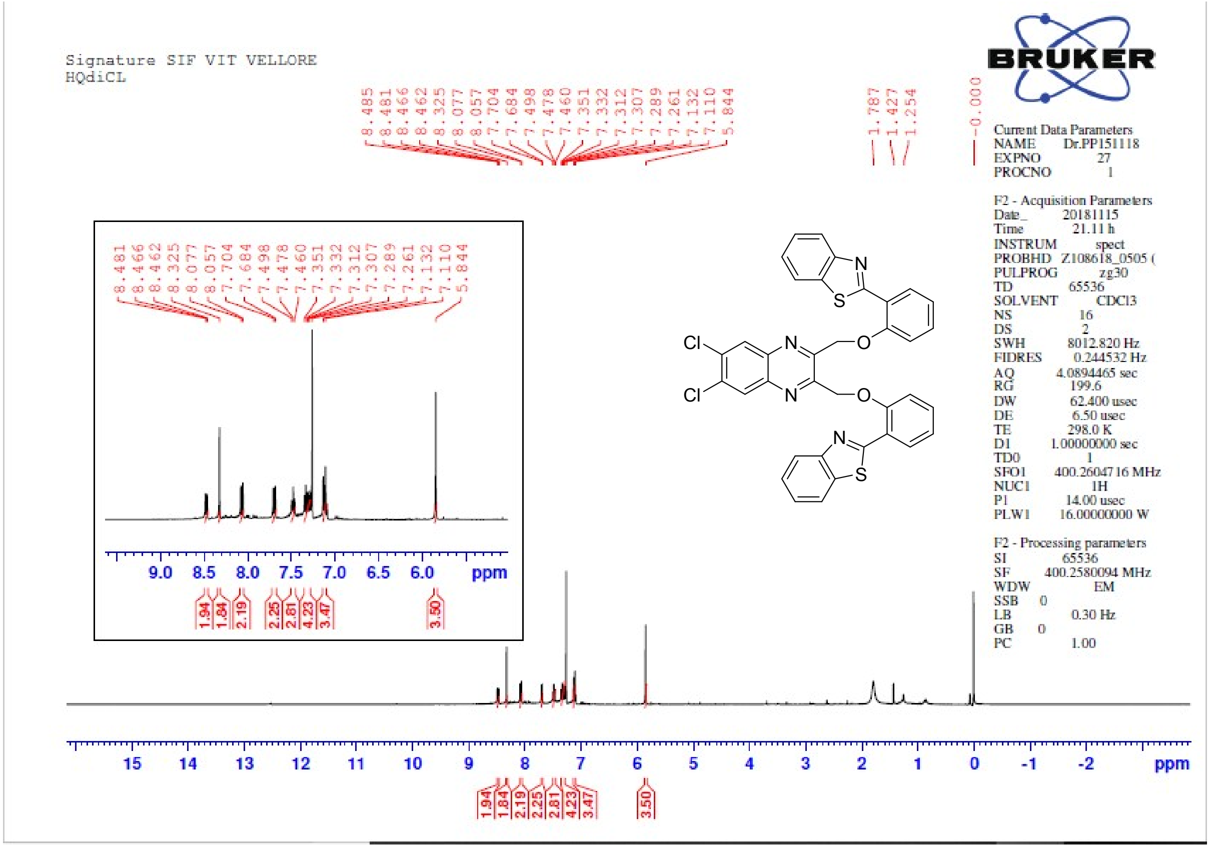
Signature SIF VIT VELLORE
HQdiCL
BRUKER
Current Data Parameters
NAME Dr.PP151118
EXPNO 27
PROCNO 1
F2 - Acquisition Parameters
Date_ 20181115
Time 21.11 h
INSTRUM spect
PROBHD Z108618_0505 (
PULPROG zg30
TD 65536
SOLVENT CDCl3
NS 16
DS 2
SWH 8012.820 Hz
FIDRES 0.244532 Hz
AQ 4.0894465 sec
RG 199.6
DW 62.400 usec
DE 6.50 usec
TE 298.0 K
D1 1.00000000 sec
TD0 1
SFO1 400.2604716 MHz
NUC1 1H
P1 14.00 usec
PLW1 16.00000000 W
F2 - Processing parameters
SI 65536
SF 400.2580094 MHz
WDW EM
SSB 0
LB 0.30 Hz
GB 0
PC 1.00
Cl
Cl
N
N
O
O
N
S
N
S
ppm

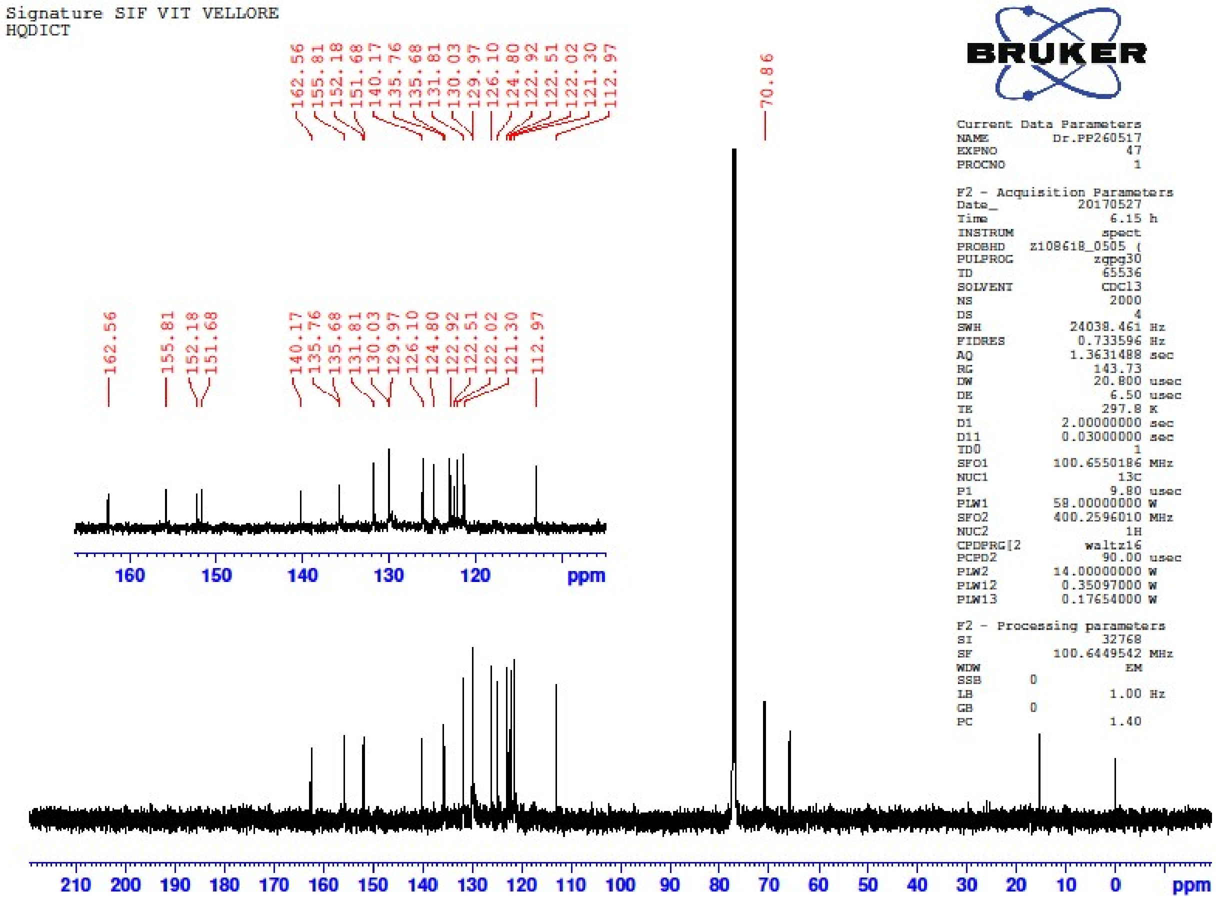
Signature SIF VIT VELLORE
HQDICT
BRUKER
162.56
155.81
152.18
151.68
140.17
135.76
135.68
131.81
130.03
129.97
126.10
124.80
122.92
122.51
122.02
121.30
112.97
70.86
Current Data Parameters
NAME Dr.PP260517
EXPNO 47
PROCNO 1
F2 - Acquisition Parameters
Date_ 20170527
Time 6.15 h
INSTRUM spect
PROBHD Z108618_0505 (
PULPROG zgpg30
TD 65536
SOLVENT CDCl3
NS 2000
DS 4
SWH 24038.461 Hz
FIDRES 0.733596 Hz
AQ 1.3631488 sec
RG 143.73
DW 20.800 usec
DE 6.50 usec
TE 297.8 K
D1 2.00000000 sec
D11 0.03000000 sec
TD0 1
SFO1 100.6550186 MHz
NUC1 13C
P1 9.80 usec
PLW1 58.00000000 W
SFO2 400.2596010 MHz
NUC2 1H
CPDPRG[2 waltz16
PCPD2 90.00 usec
PLW2 14.00000000 W
PLW12 0.35097000 W
PLW13 0.17654000 W
F2 - Processing parameters
SI 32768
SF 100.6449542 MHz
WDW EM
SSB 0
LB 1.00 Hz
GB 0
PC 1.40
ppm

^{13}C NMR of compound**7e**

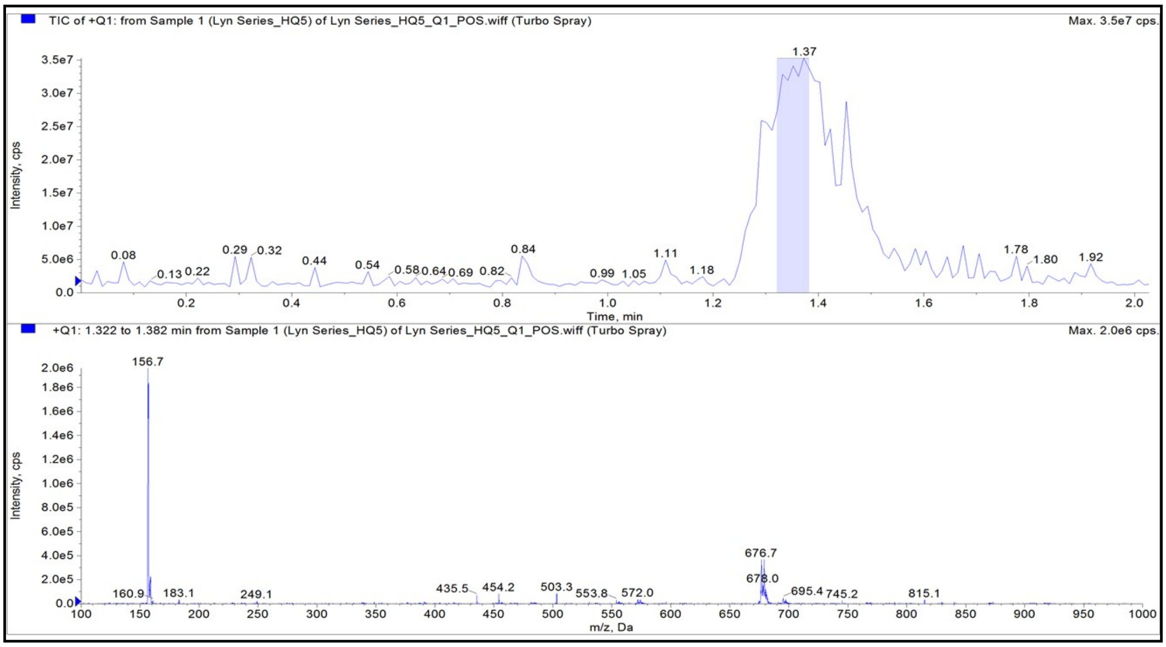

LCMS of compound **7e**

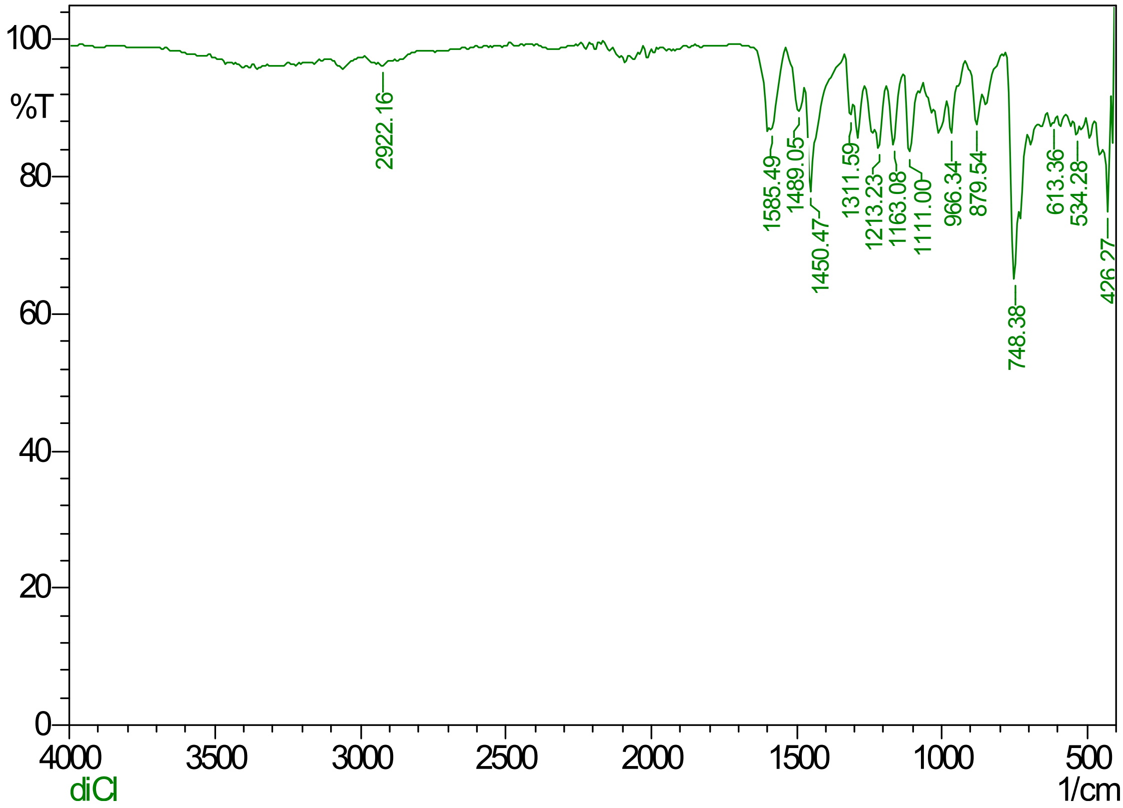

IR of compound **7e**

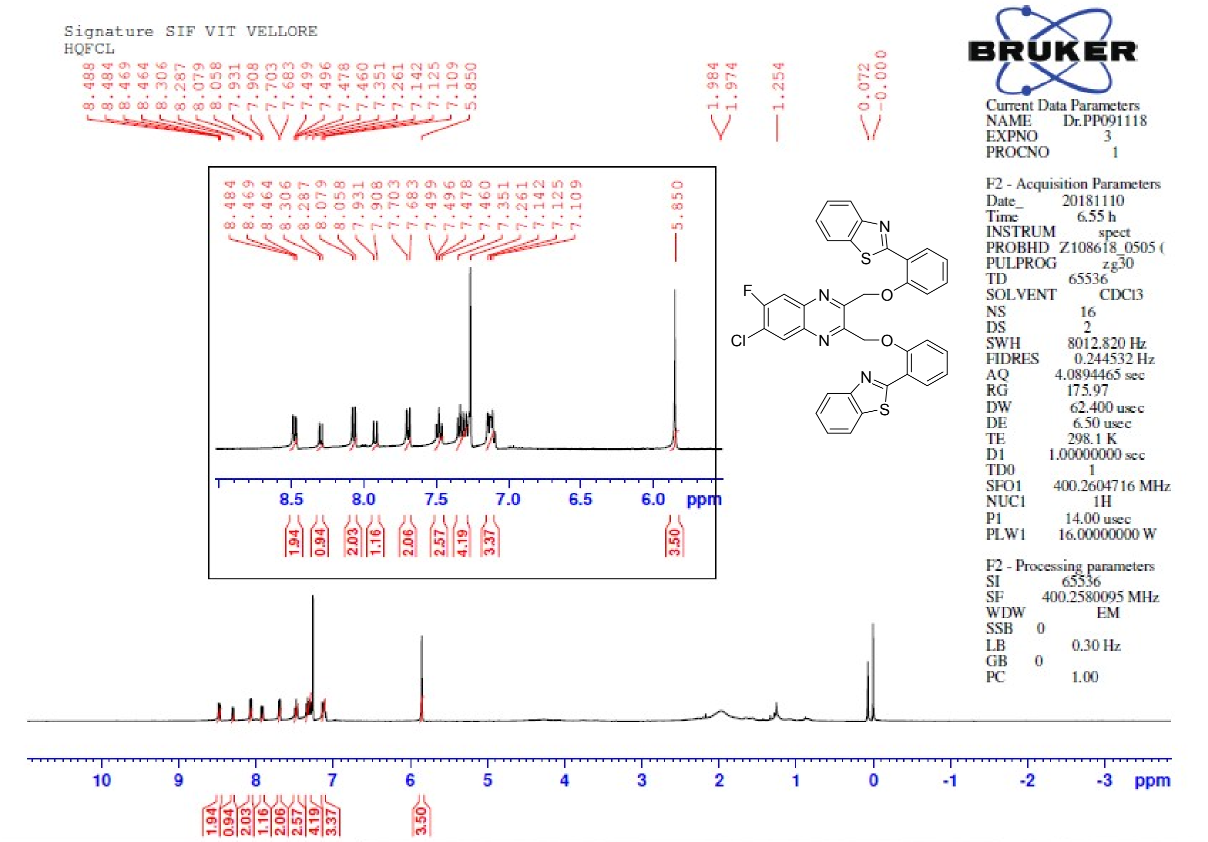

^{1}H NMR of compound **7f**

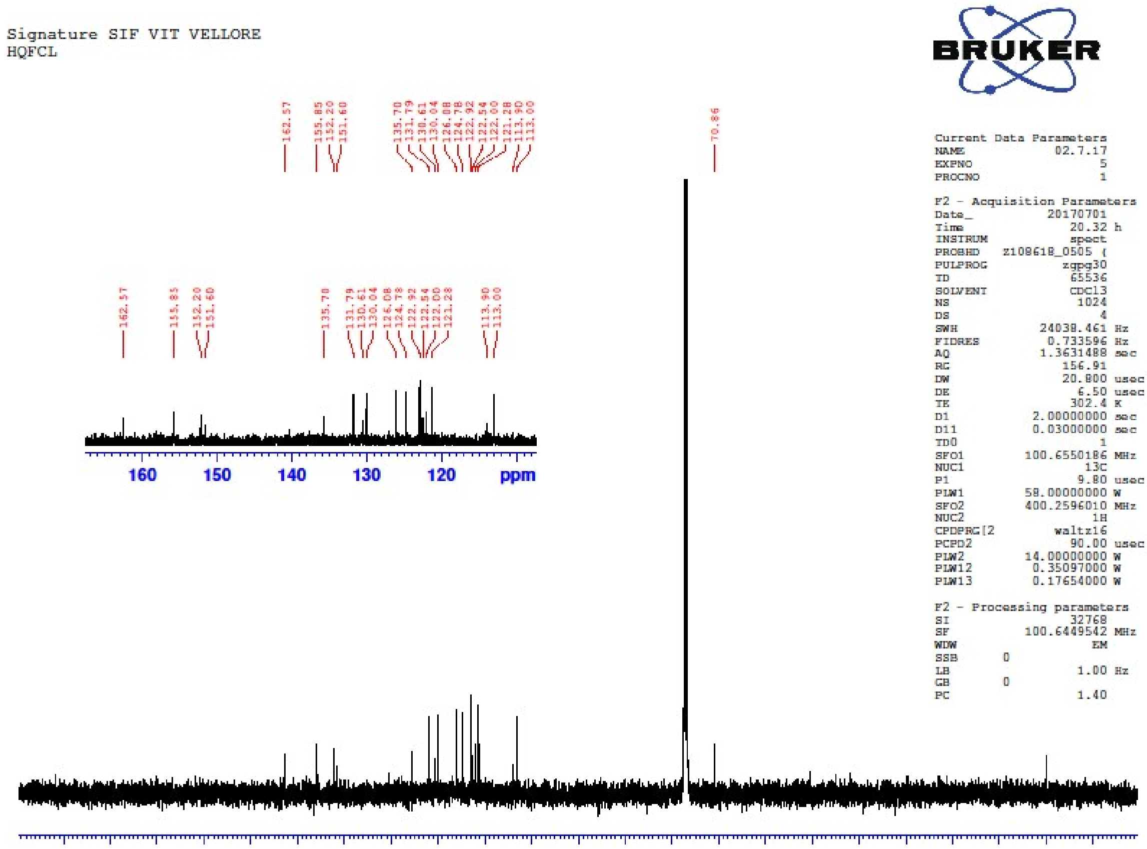

^{13}C NMR of compound **7f**

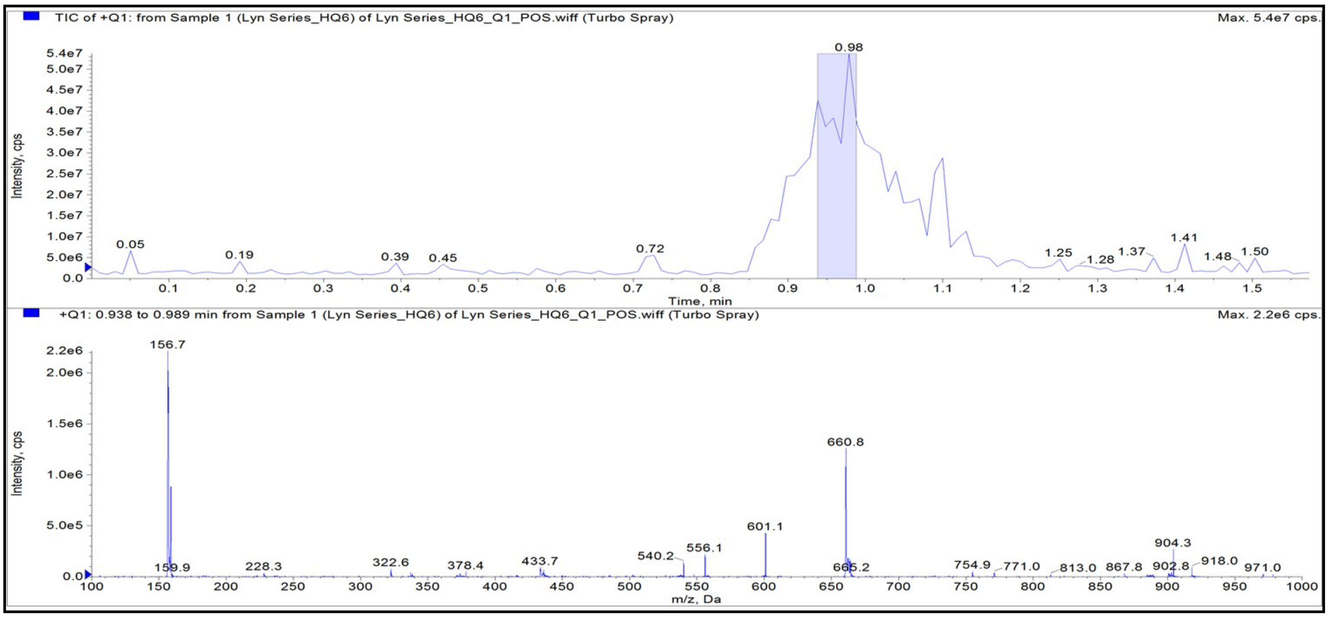

LCMS of compound **7f**

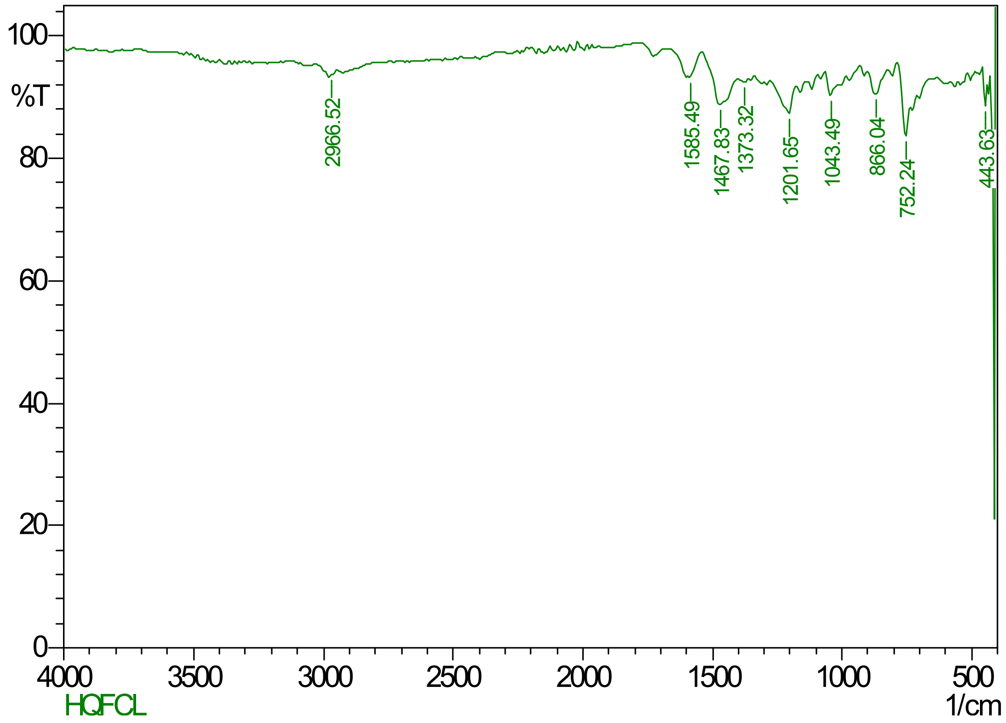

IR of compound **7f**

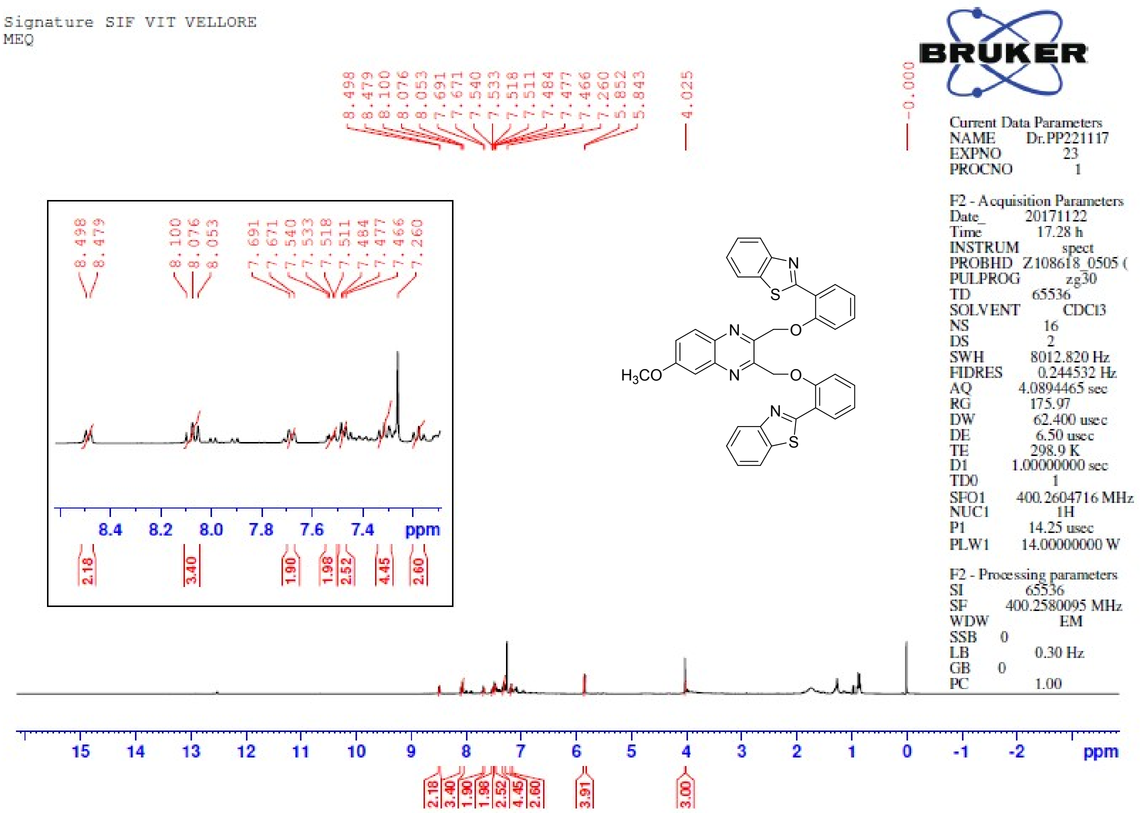

^{1}H NMR of compound **7g**

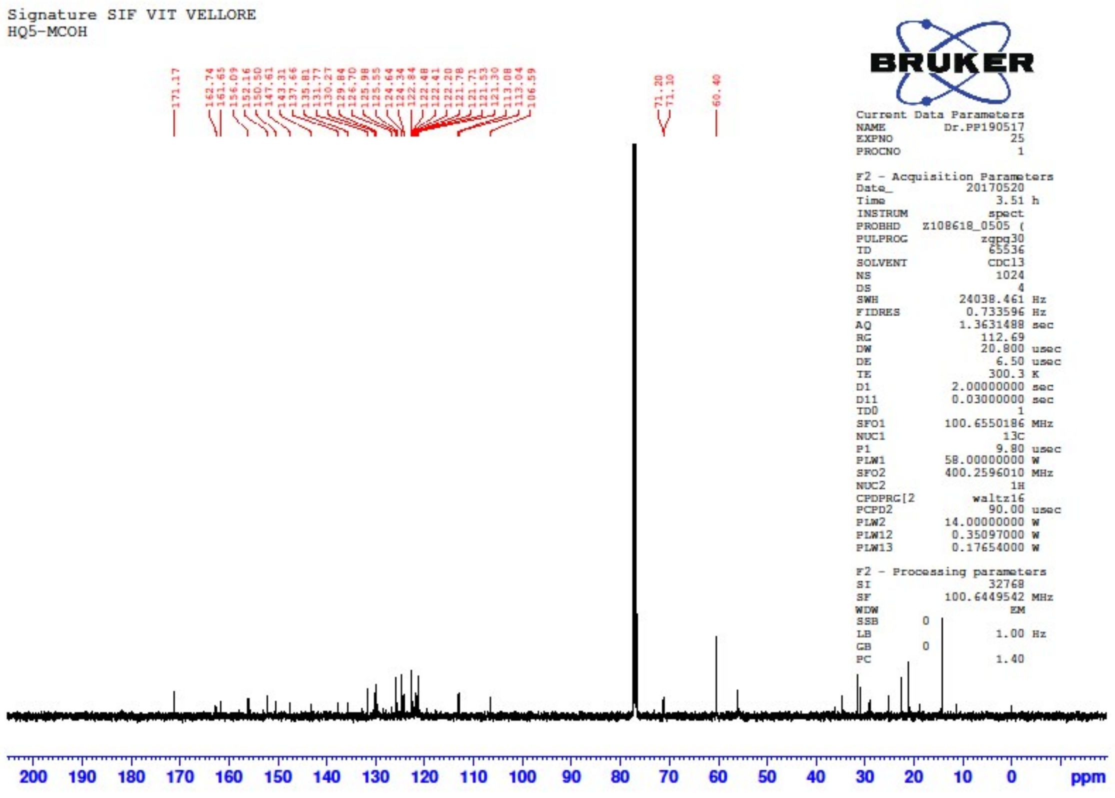

^{13}C NMR of compound **7g**

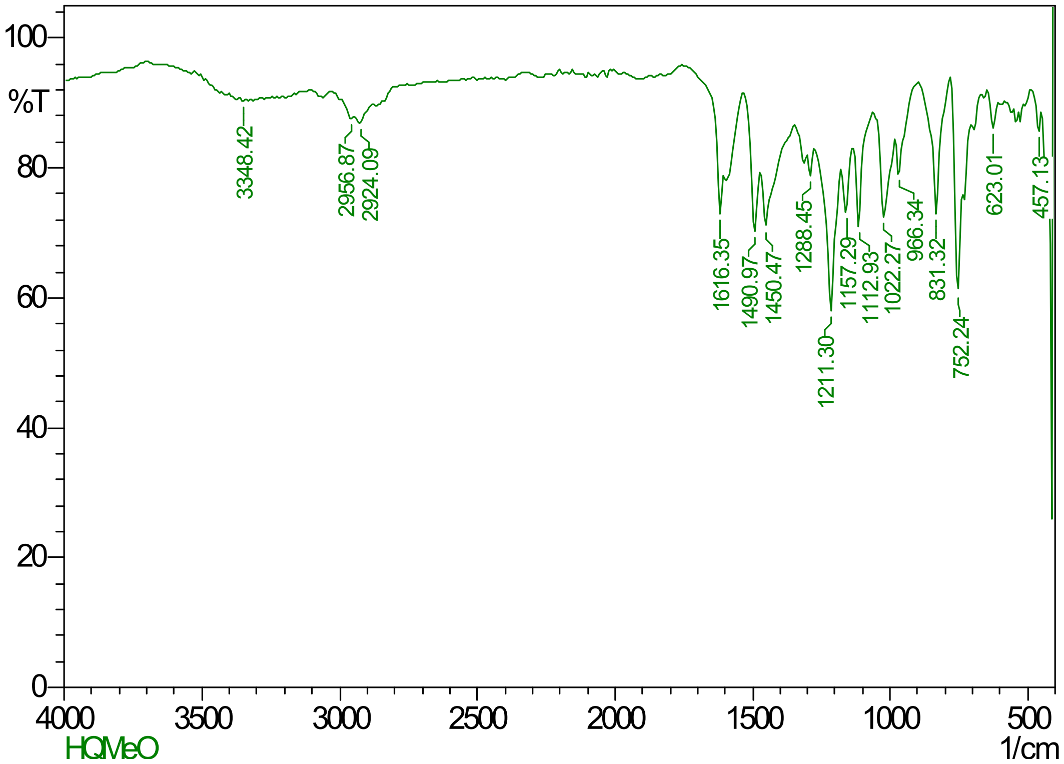

IR of compound **7g**

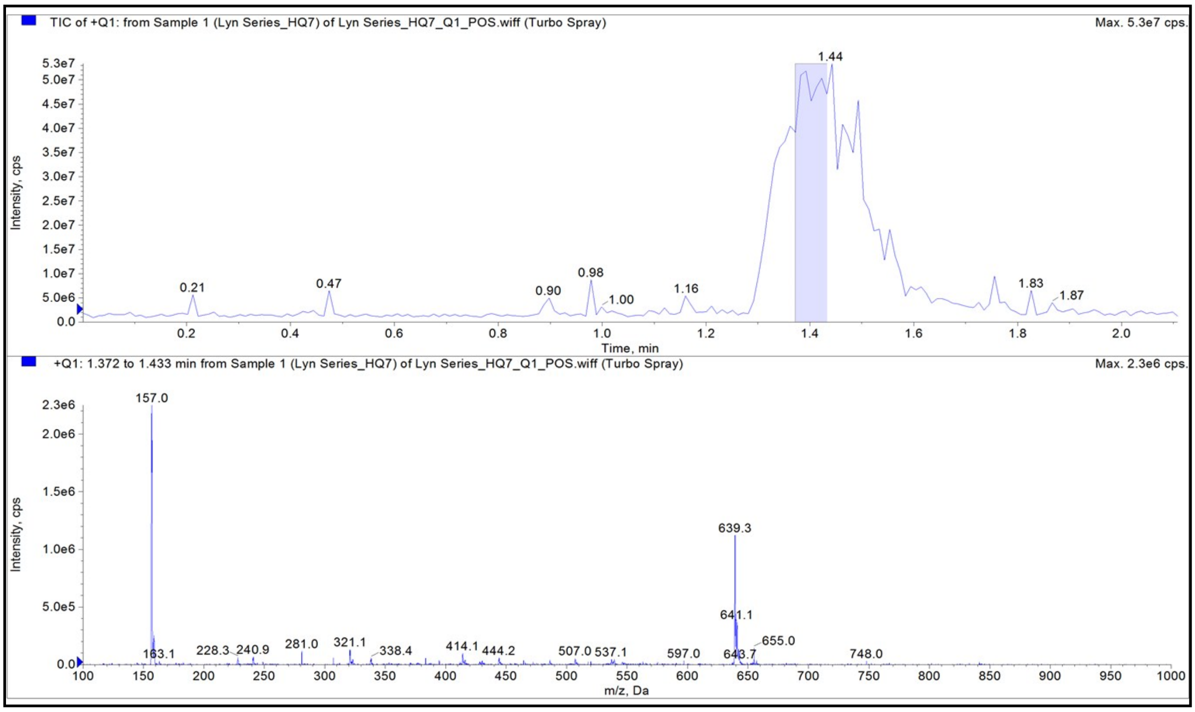

LCMS of compound **7g**

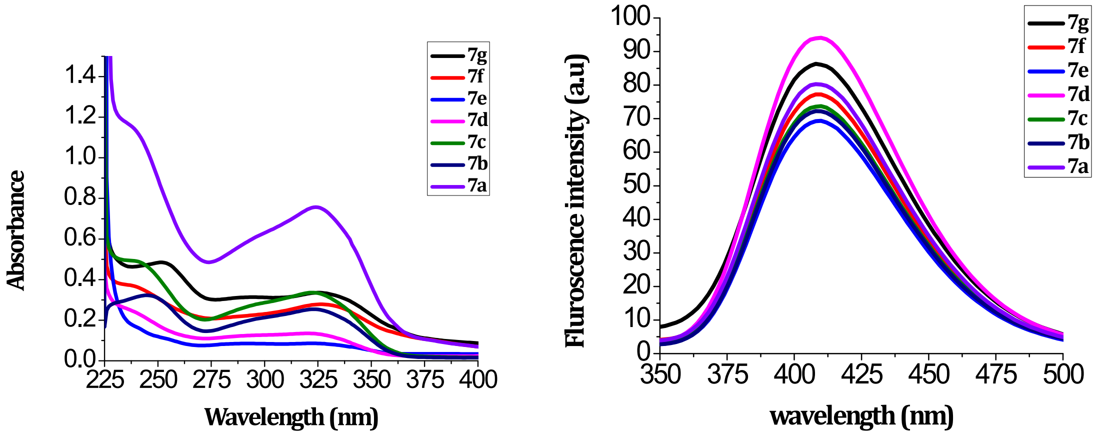

(a) (b)

Fig. S1 (a) UV and (b) fluorescence study of compound 7a-g.

Table S1 Spectroscopic data for (7a-g) at 298 K in water.

Entry	λ_{max}^{a} (nm)	λ_{max}^{b} (nm)	Stokes Shift (nm)	OD[c]	I[d]	Φ^{e}
**7a**	322	409	87	0.75	17459	0.006
**7b**	323	409	86	0.24	16149	0.017
**7c**	323	409	86	0.23	15929	0.017
**7d**	322	409	87	0.13	19952	0.04

**7e**	322	409	87	0.08	13243	0.04
**7f**	328	409	81	0.28	16315	0.014
**7g**	330	409	79	0.33	17430	0.013
Quinine[f] sulphate	350	452	102	0.029	63905	0.57

[a]abs = absorbance; [b]em = emission; [c]OD = excited absorbance; [d]I = integral area; [e]Φ = emission of quantum yield. [f]Quinine Sulphate is used as a standard for calculating the emission of quantum yield. We have used 0.5 M H_2SO_4 and water as solvent for the reference and synthesized compounds respectively.

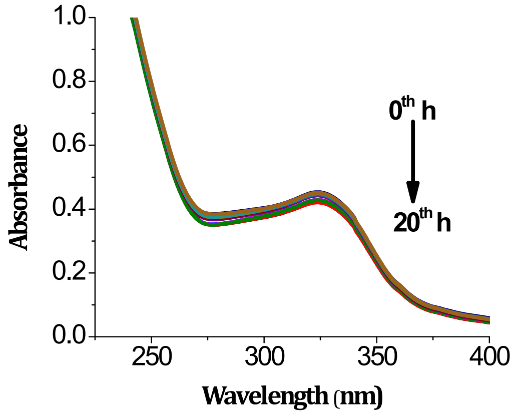

(a)

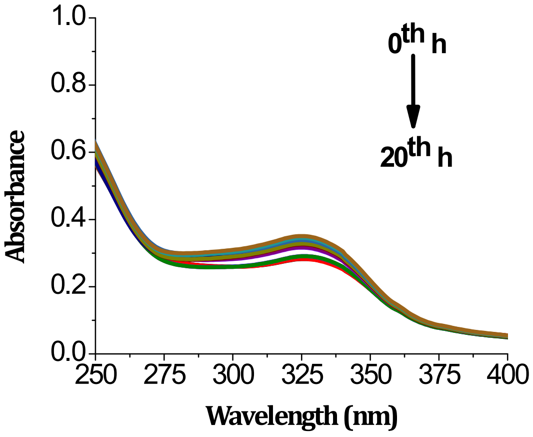

(b)

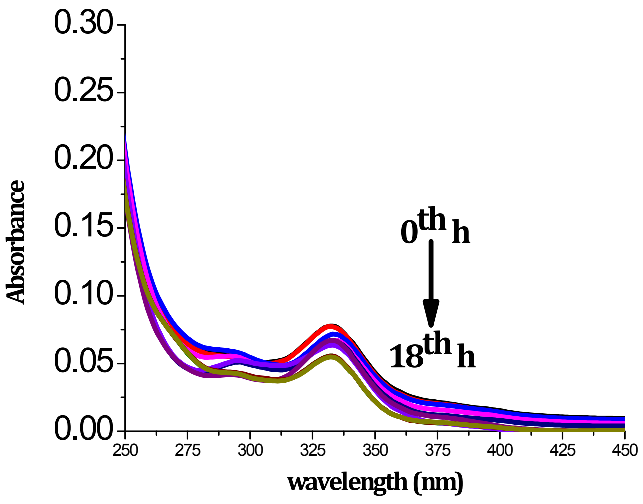

(c)

Fig. S2 Stability of compound 7e in (a) water (b) GSH (c) MTT (10% DMSO in PBS buffer)

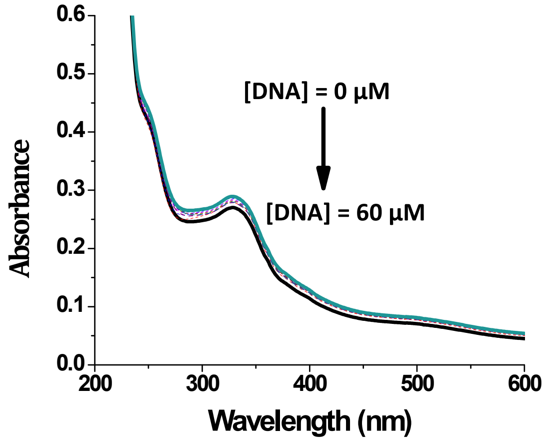

(a)

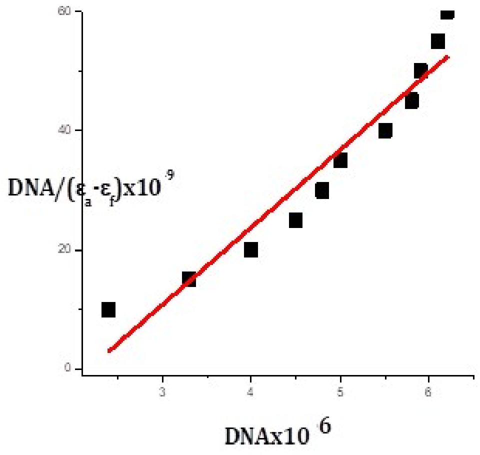

(b)

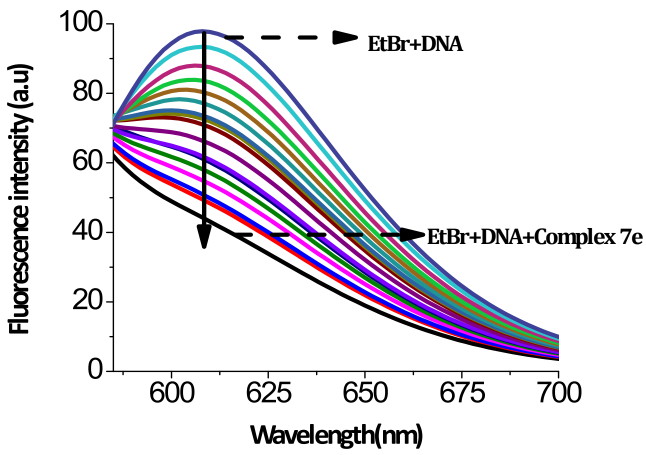

(c)

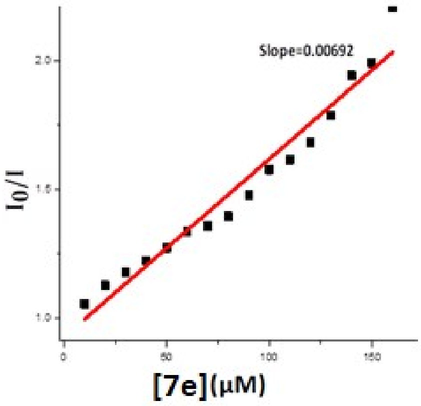

(d)

Figure S3 Ct-DNA titration plot of compound **7e** with DNA. Plot of absorbance versus wavelength (nm) for (a) compound **7e**. Plot of [DNA]/ (E_a-E_f) versus [DNA] for (b) compound **7e**. UV-visible absorption spectra of **3e** with ctDNA in PBS buffer (0.01M in pH 7.4). [**7e**] = 20μM; Variable [ctDNA] = 0-60 μM; T 298 K. Emission spectral traces of the ethidium bromide bound DNA with increasing concentration of complex (c) **7e** in 5mM Tris HCl/NaCl buffer of pH 7.2. (d) Stern-Volmer plots of F_0/F vs. Compound **7e**. λ_{ex} = 485 nm, λ_{em} = 600 nm, [DNA] = 120 μM, [EtBr] = 8 μM, [**7e**]$_{50}$ = 140 μM.

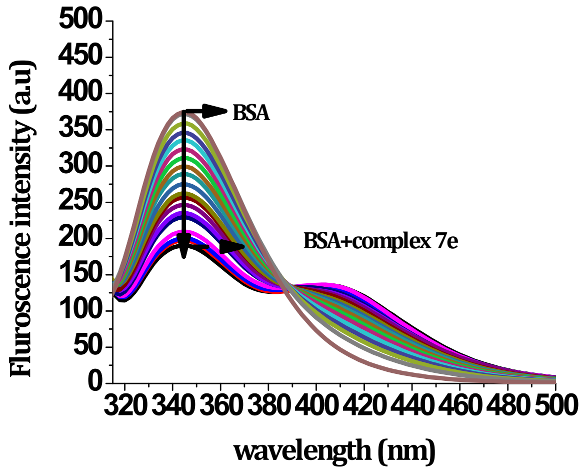

(a)

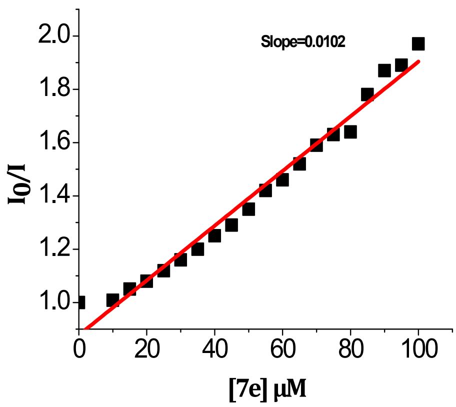

(b)

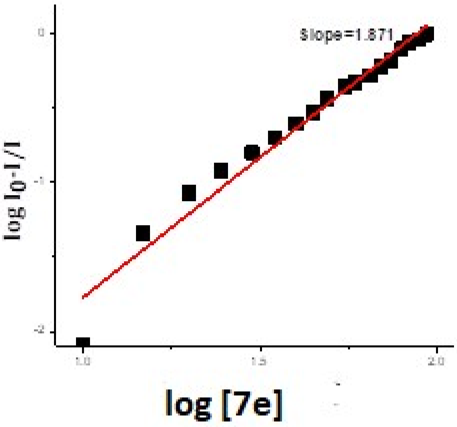

(c)

Figure S4 (a) Fluorescence emission spectra for free BSA and its fluorescence quenching with the addition of quencher **7e** (10-100μM) in 5 mM Tris HCl/NaCl buffer at pH 7.2 at 298 K (λex = 280; λem = 390 nm). (b) stern-volmer plot for BSA and quencher **7e** **(c)** logarthimic plot interaction of BSA and **7e** (Scatchard plot)

Table S2 Molecular ion, daughter ion and MRM parameters optimization

ID	Compound 7g
**Q1**	639.30
**MS2**	412.40
**DP**	25
**CE**	20

**CXP**	10
**Q1-molecular ion, MS2-daughter ion, DP-declustering potential, CE-collision energy and CXP-collision cell exit potential**	

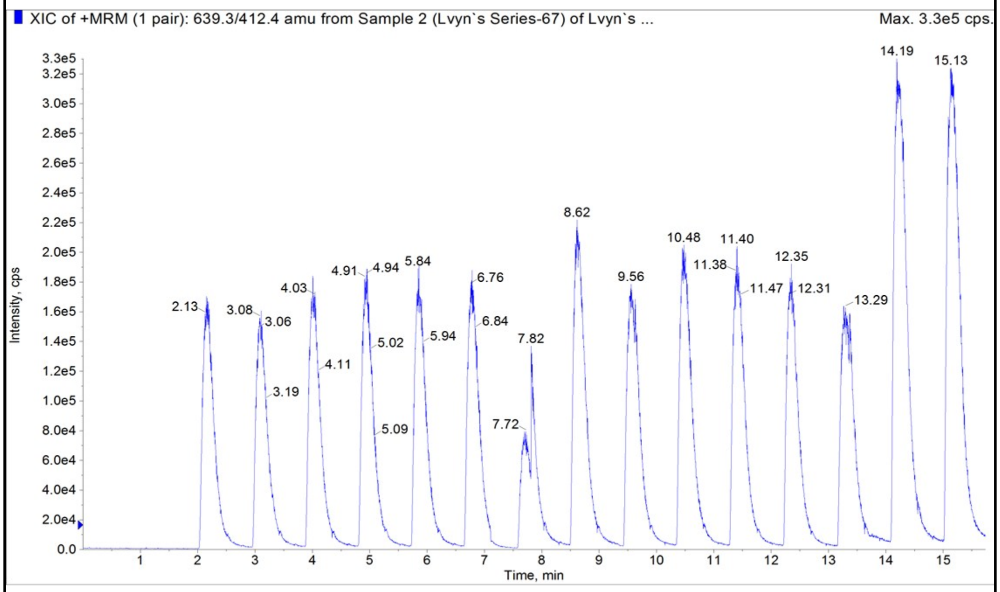

(a)

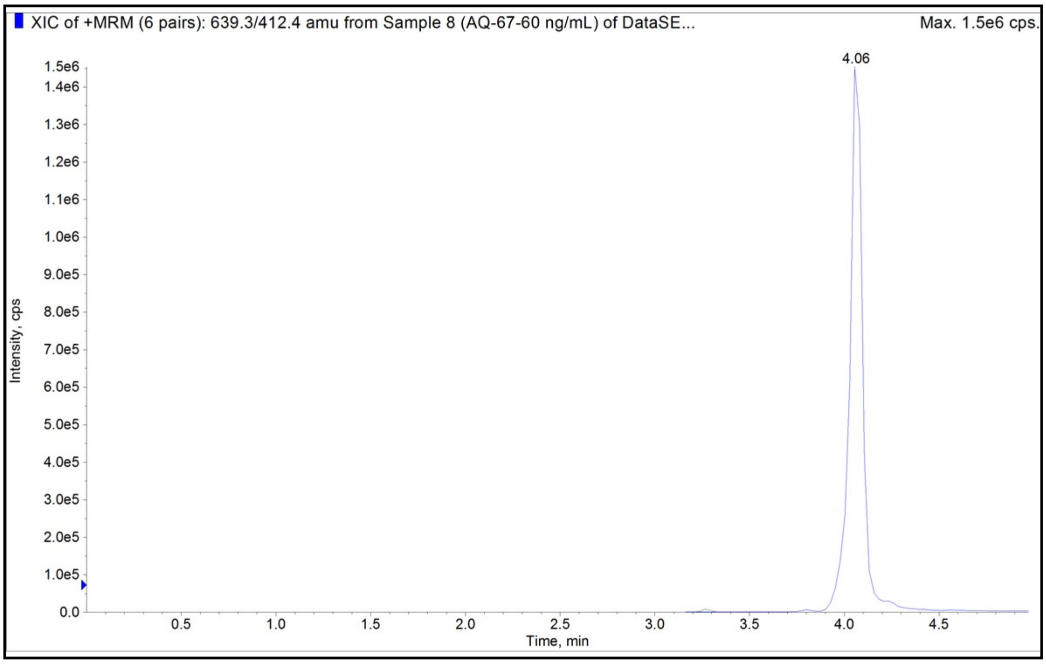

Figure S5a & S5b: Chromatogram for compound 7g

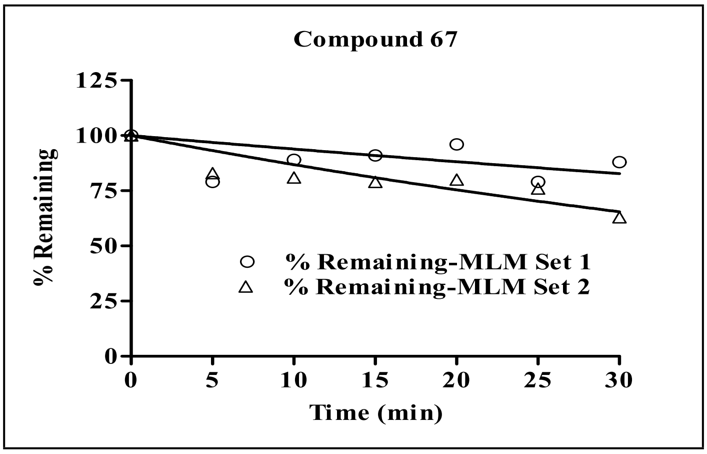

(a)

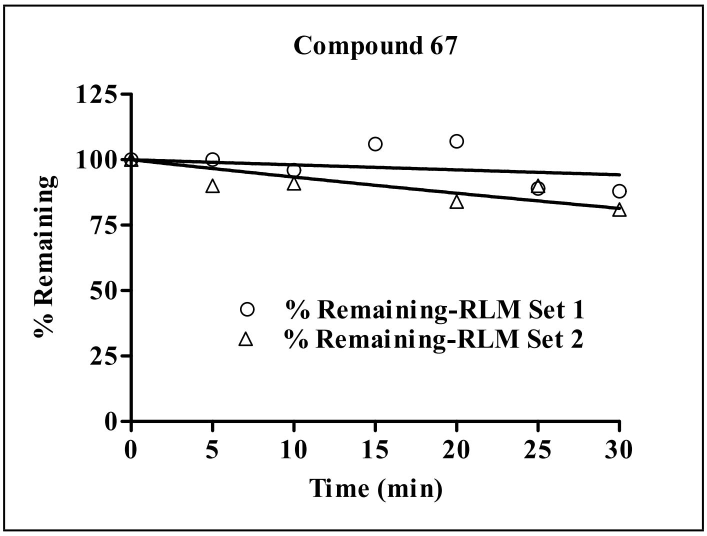

(b)

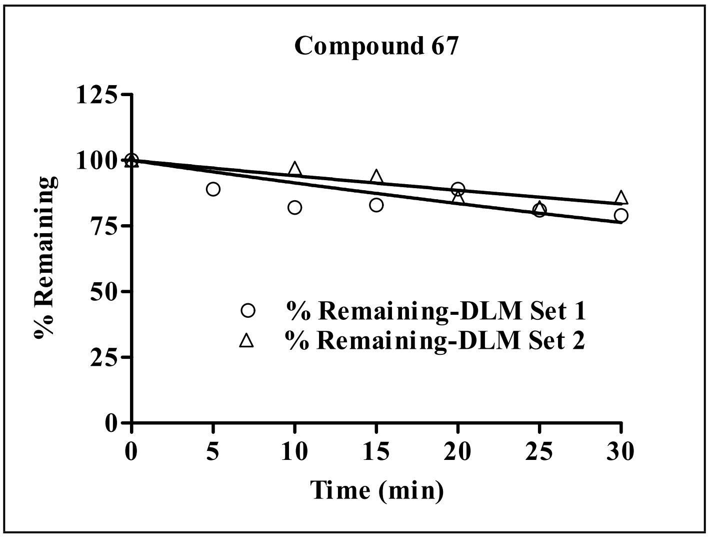

(c)

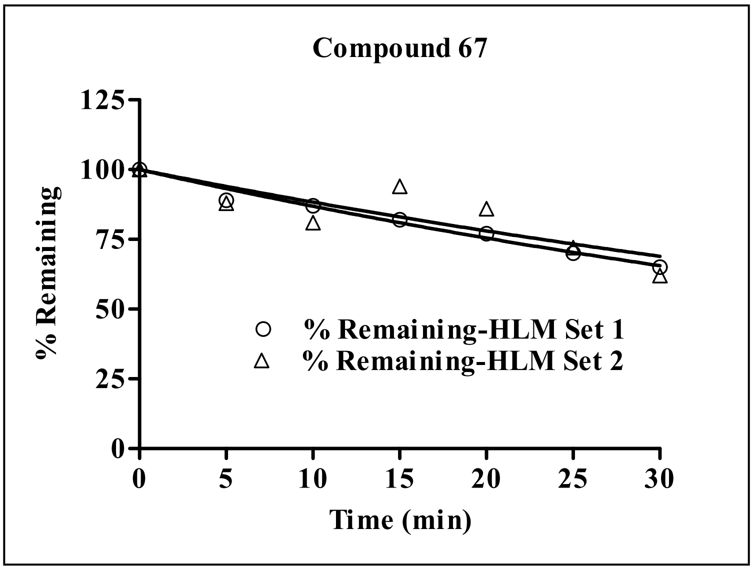

(d)

Figure S6 (a) Metabolism in mouse liver microsomes (MLM) (b) Metabolism in rat liver microsomes (RLM) (c) Metabolism in dog liver microsomes (DLM) (d) Metabolism in human liver microsomes (HLM)

References:

1) M.F. Reichmann, S.A. Rice, C.A. Thomas, P. Doty, J. Am. Chem. Soc. 76 (1954) 3047–3053
2) Tam VK, Liu Q, Tor Y. Extended ethidium bromide analogue as a triple helix intercalator: synthesis, photophysical properties and nucleic acids binding. Chem. Commun (Camb) 2006;25:2684–2686.
3) Howe Grant M, Wu KC, Baue WR, Lippard S. Binding of platinum and palladium metallointercalation reagents and antitumor drugs to closed and open DNAs. Biochem. 1976;15:4339–4346.
4) Amrita Banerjee, Jasdeep Singh, Dipak Dasgupta. Fluorescence Spectroscopic and Calorimetry Based Approaches to Characterize the Mode of Interaction of Small Molecules with DNA. J Fluoresc (2013) 23:745–752.
5) B. C. Baguley and M. LeBret, Biochemistry, 1984, 23, 937–943.
6) M. Peng, S. Shi, Y. Zhang, Spectrochim. Acta Part A Mol. Biomol. Spectrosc. 85 (2012) 190–197.
7) A. Ray, B.K. Seth, U. Pal, S. Basu, Spectrochim. Acta Part A Mol. Biomol. Spectrosc. 92 (2012) 164–174.
